# Evaluation of the clinical value of 10 estimating glomerular filtration rate equations and construction of a prediction model for kidney damage in adults from central China

**DOI:** 10.3389/fmolb.2024.1408503

**Published:** 2024-06-13

**Authors:** Xian Wang, Xingcheng Xu, Yongsheng Wang, Lei Liu, Ying Xu, Jun Liu, Benjin Hu, Xiaowei Li

**Affiliations:** ^1^ Department of Nephrology, Anhui Medical University, Fuyang People’s Hospital of Anhui Medical University, Fuyang, Anhui, China; ^2^ Center for Scientific Research, Anhui Medical University, Fuyang People’s Hospital of Anhui Medical University, Fuyang, Anhui, China; ^3^ School of Computer and Information Engineering, Fuyang Normal University, Fuyang, Anhui, China; ^4^ Department of Nephrology, The First Affiliated Hospital of Bengbu Medical College, Bengbu, Anhui, China; ^5^ Department of Nuclear Medicine, Anhui Medical University, Fuyang People’s Hospital of Anhui Medical University, Fuyang, Anhui, China; ^6^ Health Management Center, Anhui Medical University, Fuyang People’s Hospital of Anhui Medical University, Fuyang, Anhui, China

**Keywords:** glomerular filtration rate, equation, renal injure, adult, machine learning

## Abstract

**Objectives:**

This study aimed to evaluate 10 estimating glomerular filtration rate (eGFR) equations in central China population and construct a diagnostic prediction model for assessing the kidney damage severity.

**Methods:**

The concordance of 10 eGFR equations was investigated in healthy individuals from central China, and their clinical effectiveness in diagnosing kidney injury was evaluated. Subsequently, relevant clinical indicators were selected to develop a clinical prediction model for kidney damage.

**Results:**

The overall concordance between CKD-EPI_ASR-Scr_ and CKD-EPI_2021-Scr_ was the highest (weightedκ = 0.964) in healthy population. The CG formula, CKD-EPI_ASR-Scr_ and CKD-EPI_2021-Scr_ performed better than others in terms of concordance with referenced GFR (rGFR), but had poor ability to distinguish between rGFR < 90 or < 60 mL/min·1.73 m^2^. This finding was basically consistent across subgroups. Finally, two logistic regression prediction models were constructed based on rGFR < 90 or 60 mL/min·1.73 m^2^. The area under the curve of receiver operating characteristic values of two prediction models were 0.811 vs 0.846 in training set and 0.812 vs 0.800 in testing set.

**Conclusion:**

The concordance of CKD-EPI_ASR-Scr_ and CKD-EPI_2021-Scr_ was the highest in the central China population. The Cockcroft-Gault formula, CKD-EPI_ASR-Scr_, and CKD-EPI_2021-Scr_ more accurately reflected true kidney function, while performed poorly in the staging diagnosis of CKD. The diagnostic prediction models showed the good clinical application performance in identifying mild or moderate kidney injury. These findings lay a solid foundation for future research on renal function assessment and predictive equations.

## 1 Introduction

Renal injury, particularly chronic kidney disease (CKD), often exhibits a concealed onset and progression process. Precise assessment of renal function is crucial for the prevention and treatment of kidney injury. The gold standard for evaluating renal excretion function is the use of exogenous filtration biomarkers such as inulin and iodohexanol to detect glomerular filtration rate (GFR) (Selistre et al., 2016). Renal dynamic imaging technology using ^99m^Tc is currently a widely used method that can more realistically reflect renal function, and is frequently used as rGFR (Iversen et al., 2023). Unfortunately, the above detection methods have many experimental limitations and cannot dynamically observe the patient’s renal function.

In clinical practice, eGFR is a more practical approach. The Cockcroft-Gault (CG) formula1 (Cockcroft and Gault, 1976) established in 1976, has been widely adopted in China for its simplicity (Li et al., 2022; Wu et al., 2022). In 1999, the Modification of Diet in Renal Disease Research Group (MDRD) in the United States developed a series of GFR evaluation equations, known as the MDRD equations (Levey et al., 1999; Ma et al., 2006). The Chronic Kidney Disease Epidemiology Collaboration equation (CKD-EPI) published in 2009 was suggested by KDIGO guidelines (Stevens et al., 2013), but it is likely to overestimate GFR (Pottel et al., 2019). In addition, its application to the elderly has been questioned, as there were few old people included during the development process. Subsequently, the organization reported a number of adjusted new equations (Delanaye et al., 2023a). In 2016, the Full Age Spectrum (FAS) equation was focused on the coherence among different age groups (Pottel et al., 2016). But it was reported to overestimate GFR in people with low creatinine or poor renal function (Pottel et al., 2017). The European Kidney Function Consortium (EKFC) has published a novel equation based on a European population (all non-black) in 2021 (Bjork et al., 2020), and Delanaye P, et al. (Delanaye et al., 2023b). Demonstrated EKFC’s performance in cohorts from Africa and Europe. However, the European Federation of Experimental Medicine (EFLM) recommends that the 2009 version of the CKD-EPI equation should still be utilized until further updates are available. This is due to the fact that the CKD-EPI2021 equation has not demonstrated clear superiority among both black and white populations in the United States and European countries (Vestergaard et al., 2022; Delanaye et al., 2023a).

This study aimed to assess the suitability of 10 eGFR equations in central China, providing evidence-based medical support for their use in specific clinical contexts. Additionally, a logistic regression (LR) model was developed using machine learning (ML) to analyze the primary factors influencing the population.

## 2 Materials and methods

### 2.1 Survey design and population selection

**FIGURE 1:**
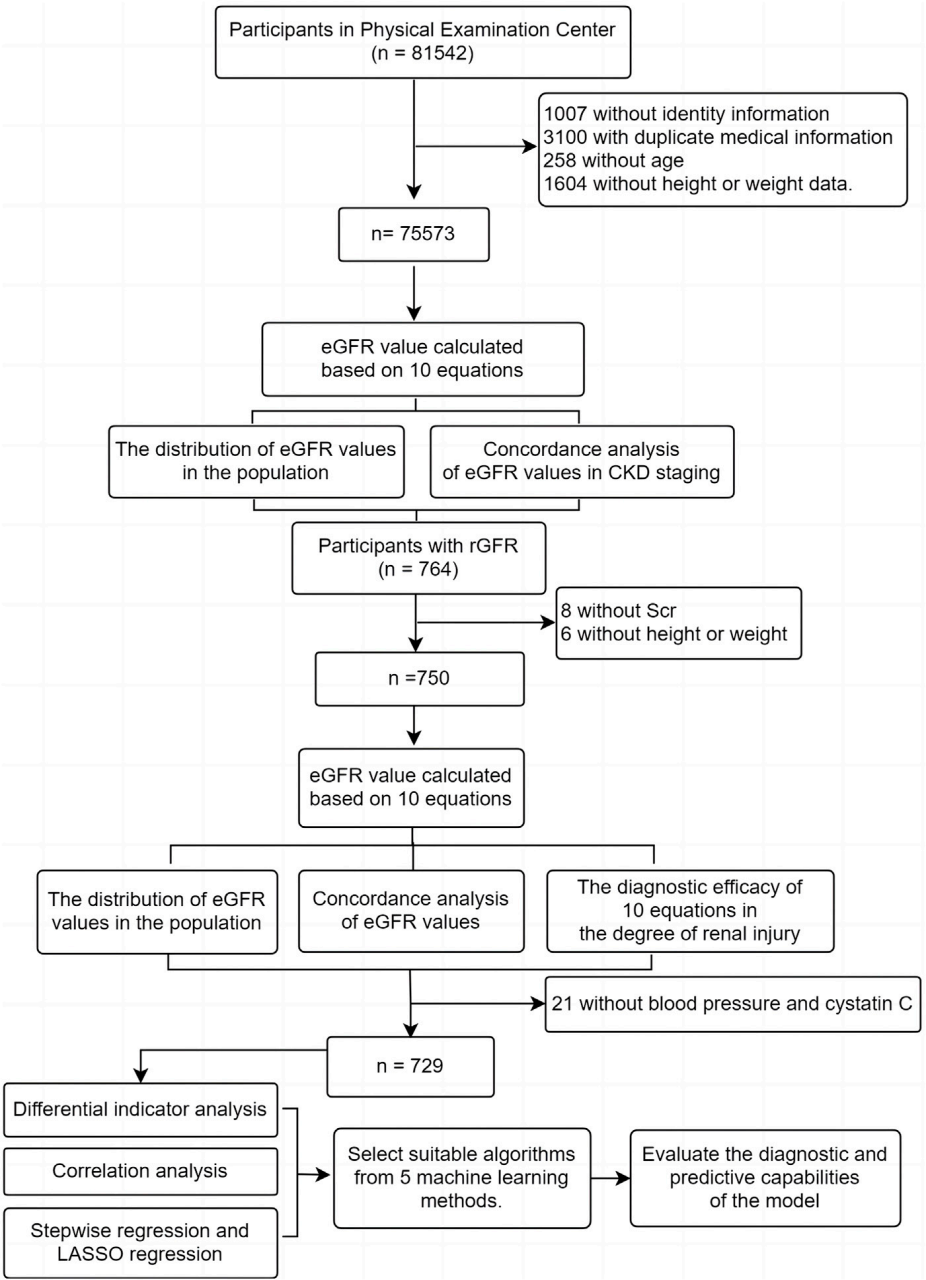
The flow chart of data analysis.

We gathered participants from first-time visitors to the Health Management Center at Fuyang People’s Hospital of Anhui Medical University from January 2016 to May 2023. Patients who underwent renal dynamic imaging examinations were also recruited from Fuyang People’s Hospital of Anhui Medical University and the First Affiliated Hospital of BengBu Medical College during the same period (Figure 1). Inclusion criteria: (Selistre et al., 2016): ≥ 18 years; (Iversen et al., 2023); Clinical examination indicators include general indicators (gender, age, height, weight) and Scr; (Cockcroft and Gault, 1976); Participants undergoing renal dynamic imaging examination also need to provide total rGFR data of both kidneys, blood pressure and serum cystatin C (Cys C); Exclusion criteria: (Selistre et al., 2016): Pregnancy; (Iversen et al., 2023); Limb deficiencies, edema, or dehydration; (Cockcroft and Gault, 1976); Previous kidney replacement therapy. This study received approval from the Ethics Review Committee of Fuyang People’s Hospital of Anhui Medical University [NO. (2019) 41].

### 2.2 Laboratory assays

Scr levels were measured using an enzymatic reaction method as reported (Sugawara et al., 2020). The current method for detecting GFR in our region relies on the renal dynamic imaging experiment using ^99m^Tc-DTPA as the developer. This study utilized this technology as the reference index for the GFR equation. The Gates’ method, executed through a Philips single photon emission computed tomography scanner, was employed to compute the rGFR.

### 2.3 Kidney function assessment criteria

Full equations available showed in Supplementary Table S1. Participants were categorized into five stages following the KDIGO guidelines (Ikizler et al., 2020): G1, ≥ 90 mL/min/1.73 m^2^; G2, 89.9–60 mL/min/1.73 m^2^; G3a, 59.9–45 mL/min/1.73 m^2^; G3b, 44.9–30 mL/min/1.73 m^2^; G4, 29.9–15 mL/min/1.73 m^2^; G5, < 15 mL/min/1.73 m^2^.

### 2.4 Model construction and ML

The data was randomly split into a training set (used for model development) and a testing set (used for model validation) in a 7:3 ratio. The Python extension package scikit-learn is utilized to develop diagnostic prediction models using various algorithms. The optimal prediction model is selected through comparison of different algorithms. Five ML models were constructed, including LR, neural network (NN); support vector machines (SVM); naive Bayes (NB); k-nearest neighbor algorithm (KNN) (Liang et al., 2020; Shi et al., 2024). In brief, LR was selected for its simplicity and interpretability; it employs L2 regularization and is a well-known method for binary classification and provides a baseline to compare more complex models. NN was chosen due to its capacity to model non-linear relationships, which is particularly beneficial given the complexity of biological systems, as it can uncover patterns that simpler models might miss. SVM were utilized for their effectiveness in high-dimensional spaces and their robustness to overfitting, especially in scenarios where the number of dimensions exceeds the number of samples. NB was included for its simplicity and performance in small datasets. Lastly, KNN was selected for its simplicity and ease of understanding; as a non-parametric method, KNN makes no underlying assumptions about the data distribution.

Model performance was assessed by comparing accuracy and prediction probability across each model. A nomogram was generated to visualize the diagnostic prediction model, and its predictive accuracy was further assessed through calibration curves and receiver operating characteristic (ROC) curves. The clinical utility of predictive models was demonstrated via decision curve analysis (DCA) and ROC curves. Net benefits in DCA were computed using the formula by Steyerberg et al. (Vickers and Elkin, 2006).

### 2.5 Statistical analysis

Data analysis was conducted using SPSS 22.0 and PASSUA. Quantitative data, assuming a normal distribution, were presented as mean ± SD/SEM, and differences between groups were assessed using the *t*-test. In cases of non-normal distribution, data was expressed as median and quartile, and group differences were examined using the Kruskal-Wallis test. Binary data were analyzed using the chi-square test. Concordance among different equations in classifying GFR stages was quantified using the weighted κ coefficient (κ), with the following cutoffs: < 0.20, poor; 0.21 to 0.40, fair; 0.41 to 0.60, moderate; 0.61 to 0.80, good; and 0.81 to 1.0, excellent. Additionally, concordance between eGFR and rGFR was assessed using Spearman correlation analysis and the intraclass correlation coefficient (ICC), where ICC < 0.4 indicated poor reliability and > 0.75 indicated good reliability. To evaluate the diagnostic effectiveness of each equation, accuracy, precision, absolute bias, and ROC curves were employed. Accuracy referred to the percentage of eGFR values falling within the range of rGFR ± 10% (P10) or ± 30% (P30). Precision was represented by the standard error (SE), and absolute bias was calculated as the absolute difference between eGFR and rGFR. Stepwise and least absolute shrinkage and selection operator (LASSO) regression analyses were utilized to identify the key indicators affecting rGFR. The threshold for statistical significance in data analysis was set at *p* < 0.05. Data visualization was conducted using Graphpad Prism 9.0. To demonstrate the consistency between two equations, Bland-Altman analysis was applied. R package was utilized for creating nomograms, calibration curves, DCA curves, and ROC curves.

## 3 Results

### 3.1 Observe the distribution of eGFR values calculated using 10 equations among the general population and the concordance between the equations

#### 3.1.1 Analysis of eGFR distribution and equation concordance in the total population

This study included a total of 75,573 participants who underwent their first health examination at Fuyang People’s Hospital from January 2016 to May 2023. The median age was 44 years, with 43,031 men (56.94%). It was important to highlight that the median BMI of the entire population was 24.08 kg/m^2^, with 29,232 (38.68%) classified as overweight and 11,739 (15.53%) as obesity. The median eGFR values calculated by equations were > 90 mL/min·1.73 m^2^ (Table 1).

**TABLE 1:** The baseline data of participants in the general population.

Characteristic	Total (*n* = 75,573)
Age (years)	44.00 (32.00, 55.00)
Age group, *n* (%)	
18–39	29,864 (39.52)
40–60	32,953 (43.60)
>60	12,756 (16.88)
Sex, *n* (%)	
Male	43,031 (56.94)
Female	32,542 (43.06)
BMI (kg/m^2^)	24.08 (21.75, 26.49)
BMI, *n* (%)	
Under weight (<18.5 kg/m^2^)	2,435 (3.22)
Normal weight (18.5–23.9 kg/m^2^)	32,167 (42.56)
Over weight (24–27.9 kg/m^2^)	29,232 (38.68)
Obesity (BMI ≥ 28 kg/m^2^)	11,739 (15.53)
Scr (mg/dL)	0.62 (0.53, 0.74)
eGFR (mL/min/1.73 m^2^)	
eGFR1	130.74 (107.61, 157.27)
eGFR2	134.82 (108.93, 168.08)
eGFR3	124.20 (100.20, 154.66)
eGFR4	166.23 (134.31, 207.25)
eGFR5	118.26 (105.26, 132.07)
eGFR6	135.94 (113.82, 164.49)
eGFR7	147.49 (111.89, 197.30)
eGFR8	111.13 (96.42, 127.54)
eGFR9	117.35 (103.89, 130.26)
eGFR10	113.36 (102.18, 123.79)

Abbreviations: years, year; BMI, body mass index (calculated as weight/height^2^); Scr, Serum creatinine: eGFR, estimated glomerular filtration rate.

**FIGURE 2:**
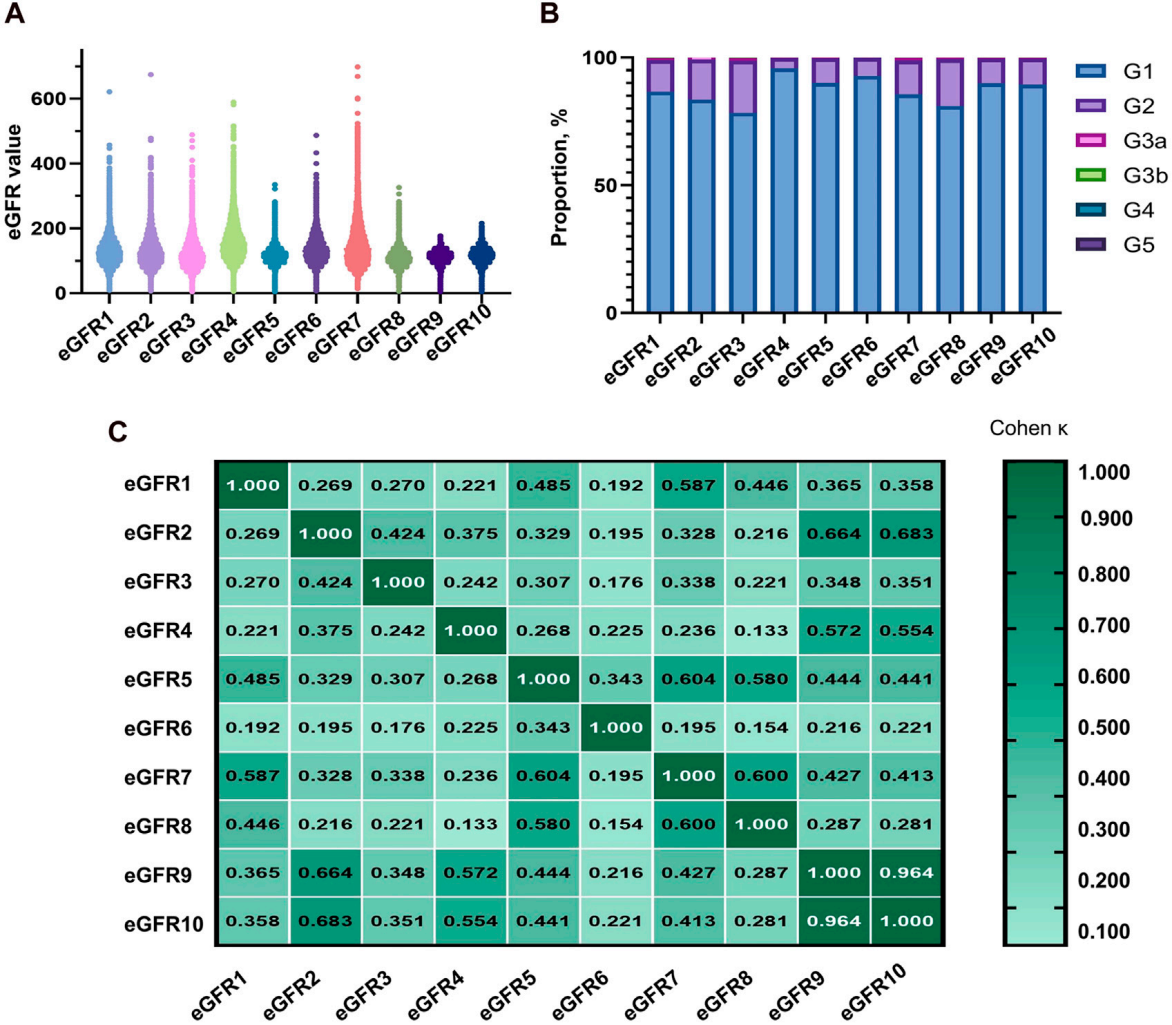
Distribution of eGFR values and concordance among equations in total populaion. **(A)** Distribution of participants according to eGFR values. **(B)** Distribution of participants across eGFR categories. **(C)** Concordance among equations in the staging of CKD. eGFR, estimated glomerular filtration rate; CKD, chronic kidney disease.

According to Figures 2A,B and Supplementary Table S2, the eGFR values calculated by each equation were primarily distributed in the G1 and G2 stages (98.69%–99.84%). Higher concordance was observed in eGFR2-eGFR9, eGFR2-eGFR10, and eGFR9-eGFR10, with weighted κ coefficients of 0.664, 0.683, and 0.964, respectively (Figure 2C; Supplementary Table S3).

#### 3.1.2 Analysis of eGFR distribution and equation concordance in subgroups

Upon analyzing the baseline data from gender groups, it was observed that the median age of males (44 years) was slightly higher than that of females (43 years), with a *p*-value of 0.000. Furthermore, there were statistically significant differences in BMI and Scr levels between the two groups. Specifically, the median eGFR value in the male group was higher than that of the female group in eGFR1, eGFR2, eGFR4, eGFR6, eGFR9, and eGFR10 (Supplementary Table S4). Most of the eGFR values were calculated using all equations in both groups, falling within the G1 and G2 phases (Supplementary Table S5). Additionally, higher concordance was observed between eGFR2-eGFR9, eGFR2-eGFR10, and eGFR9-eGFR10, irrespective of gender (Supplementary Tables S6,S7).

Regarding age composition, the proportion of males in the group aged over 60 was significantly higher (*p* = 0.000). The median BMI level in each subgroup was ≥24 kg/m^2^ and increased with age (Supplementary Table S8). The elderly group had a significantly higher proportion in the G2 stage (7.31%–62.31%) compared to other age groups (Supplementary Table S9). The highest concordances in the 18–39 years old group were eGFR9-eGFR10, eGFR5-eGFR8, and eGFR4-eGFR9 (0.990, 0.843, and 0.684, respectively). In other subgroups, the top three results of the concordance analysis were eGFR9-eGFR10, eGFR2-eGFR9, and eGFR2-eGFR10 (Supplementary Tables S10,S11).

Based on the analysis of baseline data from BMI groups, both BMI and Scr levels exhibited a similar upward trend (Supplementary Table S12). In the underweight group, the distribution of eGFR calculated by eGFR1 in the G2 stage was higher than that by other equations (30.55%), as shown in Supplementary Table S13. The results indicated that eGFR9-eGFR10, eGFR2-eGFR9, and eGFR2-eGFR10 exhibited the best concordance (Supplementary Tables S14–S17).

### 3.2 Effectiveness evaluation of eGFR values calculated from 10 equations in renal function injure

#### 3.2.1 Baseline data of individuals participating in the evaluation of 10 equations

The average age of the participants was 58.00 (50.00, 68.00) years, with 415 (55.73%) being male. The population consisted mainly of middle-aged and elderly individuals, similar to the distribution of the physical examination population mentioned earlier. The median BMI was 23.88, and the majority of the population fell into the normal and overweight categories (45.87% and 37.73% respectively). The median rGFR was 66.33 (51.16, 83.10), which was lower than the values calculated by all equations (Table 2).

**TABLE 2:** The baseline data of participants with rGFR.

Characteristic	Total (*n* = 750)
Age (years)	58.00 (50.00, 68.00)
Age group, *n* (%)	
18–39	68 (9.07)
40–60	364 (48.53)
>60	318 (42.40)
Sex, *n* (%)	
Male	415 (55.73)
Female	332 (44.27)
BMI (kg/m^2^)	23.88 (21.45, 26.27)
BMI, *n* (%)	
Under weight (<18.5 kg/m^2^)	39 (5.20)
Normal weight (18.5–23.9 kg/m^2^)	344 (45.87)
Over weight (24–27.9 kg/m^2^)	283 (37.73)
Obesity (BMI ≥ 28 kg/m^2^)	84 (11.20)
Scr (mg/dL)	0.82 (0.67, 1.07)
rGFR (mL/min/1.73 m^2^)	66.33 (51.16, 83.10)
eGFR (mL/min/1.73 m^2^)	
eGFR1	82.12 (59.25, 107.09)
eGFR2	91.55 (69.23, 111.72)
eGFR3	86.14 (65.13, 105.11)
eGFR4	112.88 (85.36, 137.75)
eGFR5	90.93 (69.70, 102.92)
eGFR6	105.72 (84.33, 125.59)
eGFR7	86.45 (66.73, 108.97)
eGFR8	105.51 (81.69, 112.88)
eGFR9	96.59 (73.30,108.13)
eGFR10	96.11 (73.62, 106.08)

Abbreviations: years, year; BMI, body mass index (calculated as weight/height^2^); Scr, Serum creatinine; rGFR, reference glomerular filtration rate; eGFR, estimated glomerular filtration rate.

Regarding gender stratification, the level of Scr was significantly higher in males than in females (*p* = 0.000). When considering age stratification, it was observed that with age, the levels of Scr increased, while the rGFR values gradually decreased, consistently with the observations made by eGFR1, eGFR9, and eGFR10 (Supplementary Tables S18–S20).

#### 3.2.2 Concordance analysis of eGFR equations

##### 3.2.2.1 Concordance analysis of eGFR equations in the total population

**FIGURE 3:**
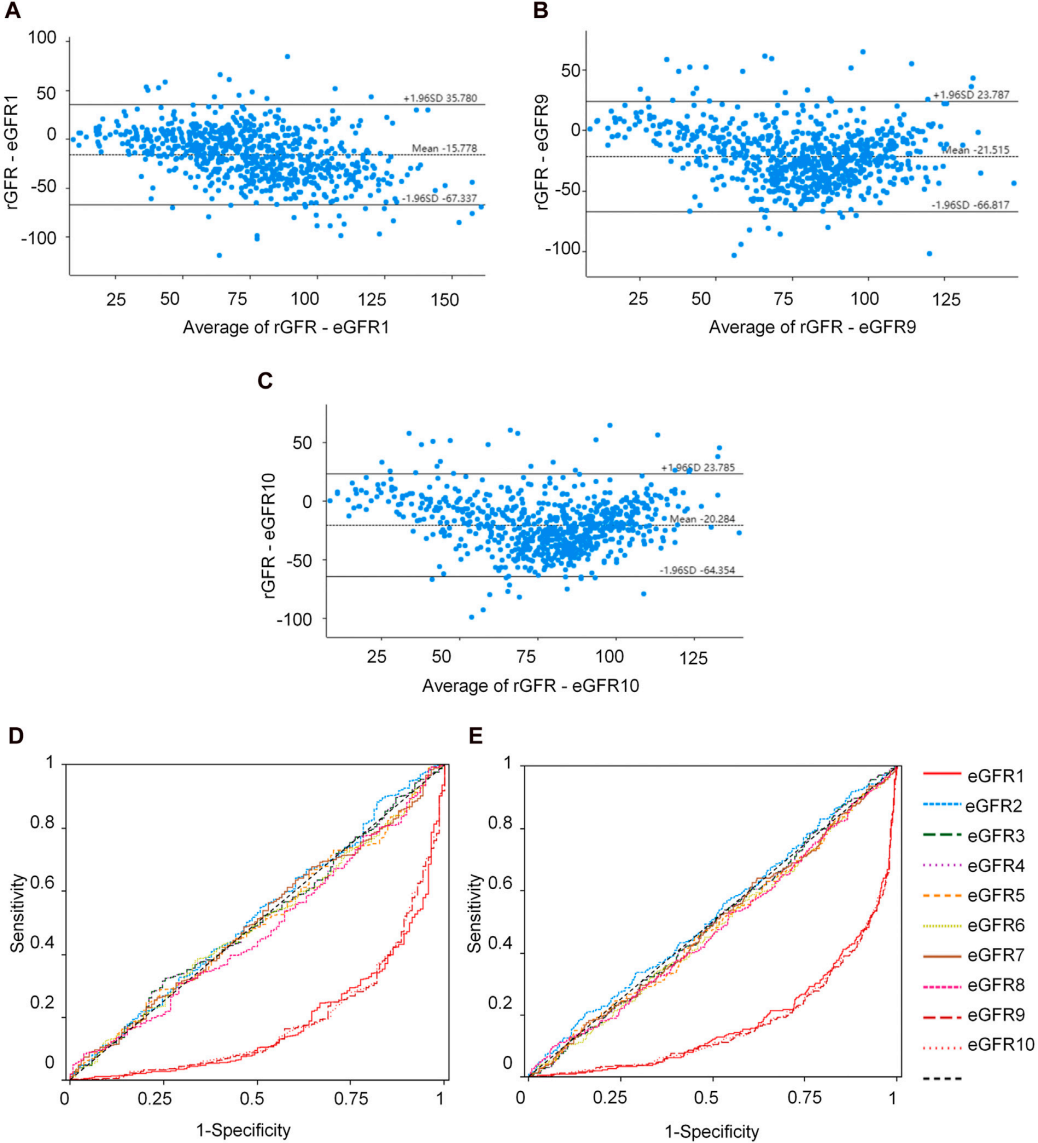
Observation of the concordance between the equation and rGFR, as well as the diagnostic efficacy of the equation in the degree of renal injury. **(A)** Bland-altman of rGFR-eGFR1. **(B)** Bland-altman of rGFR-eGFR9. **(C)** Bland-altman of rGFR-eGFR10. **(D)** ROC curves for identifying rGFR < 90 mL/min·1.73 m^2^ using different equations. **(E)** ROC curves for identifying rGFR < 60 mL/min·1.73 m^2^ using different equations. rGFR, reference glomerular filtration rate; eGFR, estimated glomerular filtration rate.

The equations with the best concordance with rGFR in the overall population included eGFR1, eGFR9 and eGFR10 (Table 3; Figures 3A–C). In the three equations, the correlation coefficients (γ) with rGFR were 0.636, 0.638 and 0.634; ICC were 0.606, 0.623 and 0.620. The weighted κ were 0.426, 0.328 and 0.327. The above three groups had the highest accuracy, with SE (30.66, 31.57 and 30.27), P10% (16.53, 14.40 and 15.60), P30% (52.13, 43.07 and 45.33), the absolute bias (18.71, 24.10, 23.16). The concordance of the above three equations was best in subgroups (Supplementary Tables S21–S29).

**TABLE 3:** Concordance analysis of eGFR equations with rGFR in the total population.

Equation	ICC (95%CI)	γ	𝛋 (95%CI)	SE	P10 (%)	P30 (%)	Absolute bias
eGFR1	0.606 (0.559 to 0.650)	0.636	0.426 (0.379 to 0.473)	30.66	16.53	52.13	18.71 (8.61, 34.96)
eGFR2	−0.081 (−0.151 to 0.009)	−0.076	−0.024 (−0.062 to 0.014)	50.56	12.13	34.40	33.10 (16.26, 56.21)
eGFR3	−0.024 (−0.095 to 0.048)	−0.020	0.007 (−0.035 to 0.050)	45.61	14.13	37.33	31.81 (14.31, 50.57)
eGFR4	−0.021 (−0.093 to 0.050)	−0.020	−0.002 (−0.036 to 0.032)	67.47	0.00	0.00	50.13 (25.40, 77.85)
eGFR5	0.010 (−0.062 to 0.081)	0.011	0.020 (−0.022 to 0.06)	40.12	12.00	36.40	30.27 (15.77, 48.39)
eGFR6	0.017 (−0.055 to 0.088)	0.021	0.015 (−0.018 to 0.049)	55.92	9.47	27.33	43.14 (20.86, 66.08)
eGFR7	−0.008 (−0.079 to 0.064)	0.002	0.020 (−0.023 to 0.063)	55.88	10.53	34.80	31.25 (16.28, 53.48)
eGFR8	0.023 (−0.049 to 0.094)	0.023	0.017 (−0.017 to 0.052)	44.77	10.80	30.40	37.04 (18.88, 53.85)
eGFR9	0.623 (0.577 to 0.665)	0.638	0.328 (0.283 to 0.373)	31.57	14.40	43.07	24.10 (11.60, 39.00)
eGFR10	0.620 (0.574 to 0.663)	0.634	0.327 (0.281 to 0.372)	30.27	15.60	45.33	23.16 (10.41, 36.53)

Abbreviations: eGFR, estimated glomerular filtration rate; SE, std. error.

##### 3.2.2.2 Diagnostic efficiency evaluation of eGFR equations

This part of the experiment first further grouped patients (1 = rGFR < 90 mL/min·1.73 m^2^). In the overall population, the highest area under the curve (AUC) values were eGFR1, eGFR9, and eGFR10 (0.181, 0.184, 0.185), with the jordan index (YI) (0.000, 0.002, 0.000), the sensitivity values (0.000, 0.002, 0.000) and the specificity values (1.000, 1.000, 1.000), as shown in Supplementary Table S30 and Figure 3D, that were similar to the results of age, sex, and BMI stratified analysis (Supplementary Tables S31–S33).

In clinical practice, diagnosing renal failure in the G3 stage is crucial as it indicates a more severe degree of kidney damage and requires special intervention measures. We categorized the population (1 = rGFR < 60 mL/min·1.73 m^2^). In the overall population, the results of ROC curve analysis showed that the highest AUC values of eGFR1, eGFR9, and eGFR10 were 0.179, 0.172, 0.173, YI values (0.000, 0.002, and 0.002), the sensitivity values (0.000, 1.000, and 1.000) and the specificity values (1.000, 0.002, and 0.002), that were similar in the subgroups (Supplementary Tables S34–37; Figure 3E).

### 3.3 Construction and validation of ML models

#### 3.3.1 Screening of differential indicators

This study further analyzed the influencing factors of rGFR in the remaining 729 participants after removing 21 missing blood pressure and Cys C data from the 750 ones mentioned above. We observed the group in two ways of grouping. The first grouping method was based on the staging of CKD, and the other two ones were based on rGFR 90 mL/min·1.73 m^2^ or 60 mL/min·1.73 m^2^ as the diagnostic threshold (CKD1, rGFR ≥ 90 or ≥ 60 mL/min·1.73 m^2^; CKD2, rGFR < 90 or < 60 mL/min·1.73 m^2^). The general characteristics of the two groups of patients were shown in the table, which can be seen that there were significant statistical differences in age, SBP, and Cys C levels between the two groups based on two different grouping methods (Table 4).

**TABLE 4:** The Baseline data of participants grouped on the severity of kidney injury.

Characteristic	rGFR ≥ 90 mL/min·1.73m^2^ (*n* = 123)	rGFR < 90 mL/min·1.73 m^2^ (*n* = 606)	Z/[χ2]	*p*-value	rGFR ≥ 60 mL/min·1.73 m^2^ (*n* = 453)	rGFR < 60 mL/min·1.73 m^2^ (*n* = 276)	Z/[χ2]	*p*-value
Sex male *n* (%)	66 (53.66)	340 (56.11)	[0.248]	0.618	244 (53.86)	162 (58.70)	[1.623]	0.203
Age (years)	50 (40.00,58.00)	60.00 (52.00,69.00)	−7.919	0.000	55.00 (48.00,65.00)	65.00 (54.00,73.00)	−7.975	0.000
Height (m)	1.67 (1.60,1.70)	1.65 (1.60,1.70)	−1.725	0.085	1.65 (1.60,1.70)	1.65 (1.60,1.70)	−1.286	0.198
weight (kg)	66.00 (56.00, 75.00)	64.00 (56.00,72.00)	−1.433	0.152	65.00 (57.00,73.00)	62.50 (55.00,71.00)	−1.334	0.182
BMI (kg/m^2^)	23.88 (21.70,26.00)	23.83 (21.30,26.20)	−0.598	0.550	23.88 (21.50,26.00)	23.73 (21.20,26.40)	−0.417	0.676
SBP (mmHg)	126.00 (115.00,140.00)	138.00 (123.00,151.30)	−4.929	0.000	132.00 (119.00,150.00)	139.50 (123.30,154.00)	−3.210	0.001
DBP (mmHg)	76.00 (69.00,84.00)	80.00 (70.00,90.00)	−2.223	0.020	79.00 (70.00,89.00)	80.00 (69.00,89.00)	−0.071	0.944
Cys C (mg/L)	0.84 (0.70,1.00)	1.15 (0.90,1.50)	−9.829	0.000	0.95 (0.80,1.10)	1.42 (1.20,1.90)	−14.913	0.000
Scr (mg/dL)	0.69 (0.60,0.80)	0.87 (0.70,1.10)	−7.372	0.000	0.74 (0.60,0.90)	1.05 (0.80,1.40)	−12.798	0.000

Abbreviations: years, year; BMI, body mass index (calculated as weight/height^2^); SBP, systolic blood pressure; DBP, diastolic blood pressure; Cys C, serum cystatin C; scr, Serum creatinine; rGFR, reference glomerular filtration rate.

#### 3.3.2 Correlation analysis between patient indicators and rGFR

**FIGURE 4:**
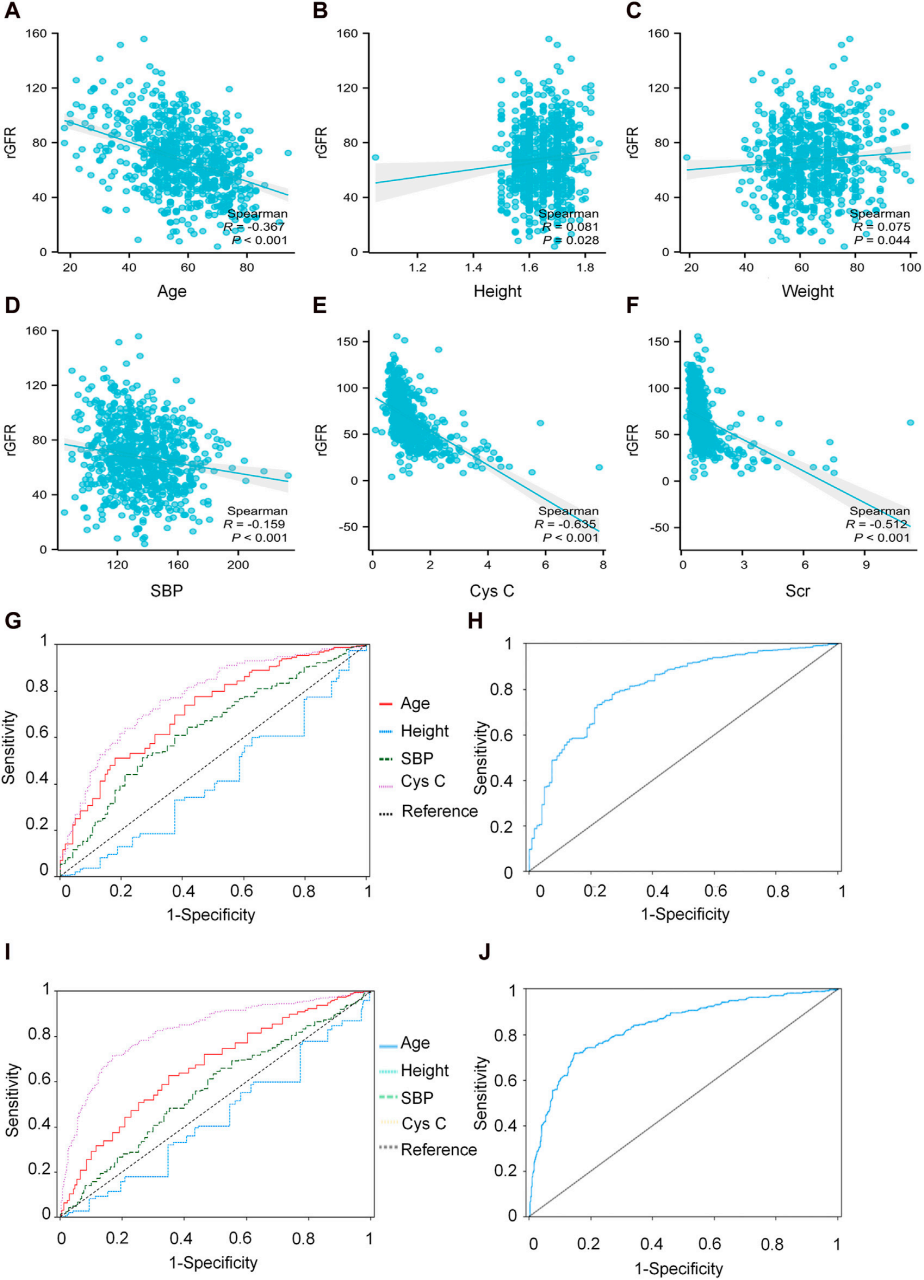
The correlation between indicators and rGFR. **(A)** The correlation coefficient between age and rGFR. **(B)** The correlation coefficient between Height and rGFR. **(C)** The correlation coefficient between weight and rGFR. **(D)** The correlation coefficient between SBP and rGFR. **(E)** The correlation coefficient between Cys C and rGFR. **(F)** The correlation coefficient between Scr and rGFR. **(G,H)** ROC curve for independent indicators and Joint diagnostic system in distinguishing rGFR < 90 mL/min·1.73 m^2^. **(I,J)** ROC curve for independent indicators and Joint diagnostic system in distinguishing rGFR < 60 mL/min·1.73 m^2^. rGFR, reference glomerular filtration rate; Cys C, serum cystatin C; Scr, Serum creatinine.

We conducted a correlation analysis to examine the relationship between age, height, SBP, Cys C, Scr, and rGFR. From Supplementary Table S38 and Figures 4A–F, the results indicated significant correlations among age, height, SBP, Cys C, Scr, and rGFR (−0.367, 0.081, −0.159, −0.635, and −0.512, respectively).

#### 3.3.3 Stepwise regression and LASSO regression observation and screening of the main indicators affecting rGFR

We employed all indicators as independent variables, with rGFR as the dependent variable. After automatic selection by the model, four essential indicators remained in the model: age, height, SBP, and Cys C. The regression coefficients for age, height, SBP and Cys C respectively were −0.455 (*t* = −7.983, *p* = 0.000), 29.662 (*t* = 3.069, *p* = 0.002), −0.082 (*t* = −2.395, *p* = 0.017), and −16.634 (*t* = −16.306, *p* = 0.000). The selected indicators were used for subsequent LASSO regression equations (Supplementary Table S39).

We utilized LASSO regression to screen influential factors in data. Age, height, SBP, and Cys C were employed as independent variables, while rGFR served as the dependent variable (Supplementary Table S39). The regression coefficients for age, height, SBP, and Cys C were −0.446 (*t* = −7.916, *p* = 0.000), 25.843 (*t* = 2.702, *p* = 0.007), −0.070 (*t* = −2.08, *p* = 0.037), −16.296 (*t* = −16.152, *p* = 0.000).

#### 3.3.4 ROC curve analysis was further used to screen indicators with diagnostic efficacy for kidney injury

To evaluate the diagnostic efficacy of factors with statistical differences in the early diagnosis of kidney disease, ROC curve analysis was conducted. The AUC values for age, SBP, and Cys C were 0.726, 0.641 and 0.781, respectively. The corresponding YI values were 0.337, 0.243 and 0.436. Sensitivity values were 0.776, 0.512 and 0.761, while specificity values were 0.561, 0.732, and 0.675.

The differential diagnostic efficacy of moderate kidney injure was evaluated using AUC values, which were 0.676, 0.571 and 0.829, with YI values (0.278, 0.145 and 0.546), the sensitivity values (0.627, 0.659 and 0.714) and the specificity values (0.651, 0.486 and 0.832).

Additionally, a combined diagnostic system was developed by incorporating differential indicators to generate ROC curves. The AUC values for this system were 0.814 and 0.833, with YI values (0.512 and 0.549), sensitivity values (0.731 and 0.728), and specificity values (0.780 and 0.821), as shown in Supplementary Table S40 and Figures 4G–J.

### 3.4 Constructing and evaluating the predictive model using ML

#### 3.4.1 Constructing and evaluating the predictive model based on rGFR < 90 mL/min·1.73 m^2^

**FIGURE 5:**
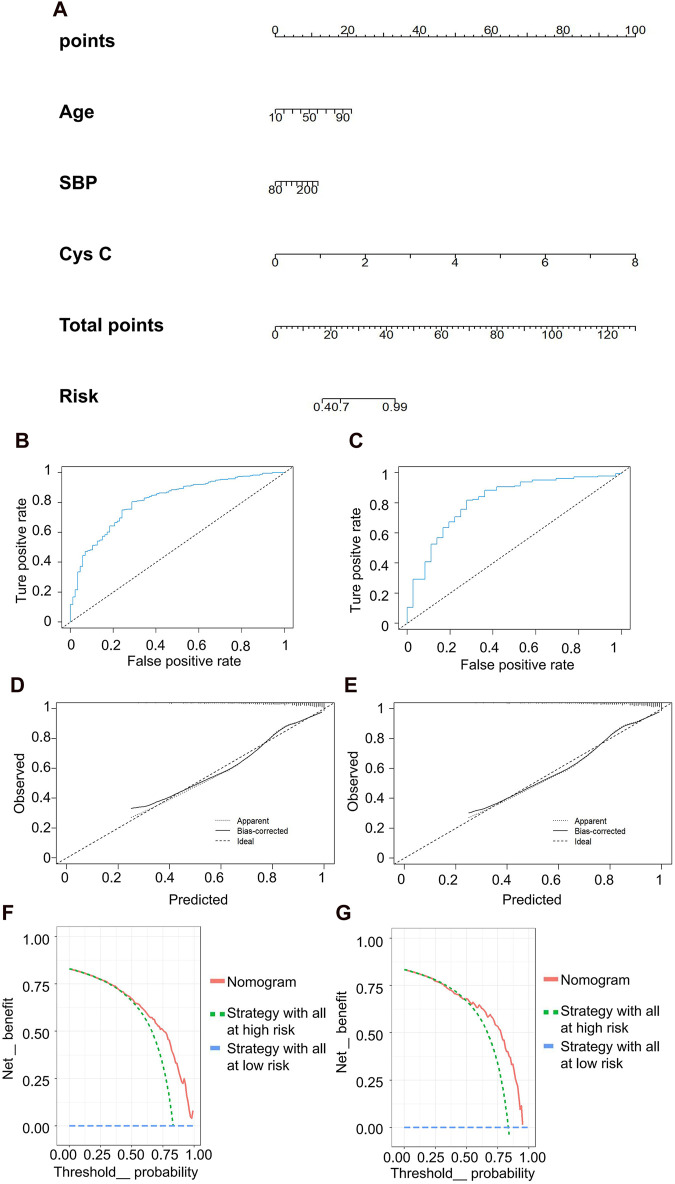
Clinical efficacy analysis for LR model based on rGFR < 90 mL/min 1.73 m^2^. **(A)** Nomogram prediction model. **(B,C)** ROC curves for training set and testing set. **(D,E)** Calibration outcome on training set and testing set. **(F,G)** DCA for training set and testing set. LR, logistic regression; rGFR, reference glomerular filtration rate; ROC, receiver operating characteristic; DCA, decision curve analysis.

The predictive model for mild kidney injury included age, SBP, and Cys C as independent variables, with rGFR 90 mL/min·1.73 m^2^ as the dependent variable. Five ML models were constructed, including LR, NN, SVM, NB and KNN. The accuracy rates achieved by these models were as follows: LR −84.932%, NN −84.018%, SVM −84.018%, NB −78.995%, and KNN −82.648%. We further used the predicted probability to draw the ROC curve to evaluate the predictive ability of the five algorithms, and the AUC value of LR was highest (Table 5). The linear predictor for the subject LR model was defined as: −5.259 + (0.048 × age) + (0.013 × SBP) + (2.393 × Cys C). The contribution of each indicator in the model to predict the occurrence of disease was shown by the nomogram, with the greatest contribution made by Cys C (Figure 5A). The classification ability of the logistic regression model in the testing set was illustrated in Figures 5B,C. The AUC, accuracy, recall rate, and F1 score for the training set were 0.811, 0.787, 0.802, and 0.862, respectively, while for the testing set, they were 0.812, 0.783, 0.796, and 0.860. The calibration curve closely approximated the 45° line, confirming a good agreement between actual and predicted values (Figures 5D,E). In DCA curve analysis, two curves were obtained as a reference for clinical practicality comparison, one assuming that all patients were positive and the other assuming they were negative cases. The clinical practicality of the model is generally considered better than the random solution when the decision curve of the model is positioned on the upper right side of the two decision curves. The DCA curves demonstrated that the predictive model derived from the nomogram is clinically useful for the modeled population (Figures 5F,G).

**TABLE 5:** Comparison of five machine learning methods.

	rGFR < 90 mL/min·1.73 m^2^					rGFR < 60 mL/min·1.73 m^2^				
	Accuracy (%)	AUC (95%CI)	YI	Sen	Spe	Accuracy (%)	AUC (95%CI)	YI	Sen	Spe
LR	84.932	0.809 (0.768–0.851)	0.516	0.809	0.707	78.082	0.834 (0.803–0.865)	0.834	0.539	0.736
NN	84.018	0.636 (0.581–0.691)	0.215	0.483	0.732	60.731	0.714 (0.675–0.752)	0.714	0.348	0.710
SVM	84.018	0.653 (0.604–0.703)	0.235	0.479	0.756	60.731	0.714 (0.675–0.752)	0.714	0.348	0.710
NB	78.995	0.693 (0.643–0.743)	0.293	0.602	0.691	66.667	0.640 (0.597–0.683)	0.64	0.273	0.457
KNN	82.648	0.798 (0.759–0.836)	0.456	0.497	0.959	63.927	0.751 (0.716–0.787)	0.751	0.384	0.790

Abbreviations: rGFR, reference glomerular filtration rate; ROC, receiver Operating Characteristic; AUC, area under the curve; LR, logistic regression; NN, neural network; SVM, support vector machines; NB, naive Bayes; KNN, k-nearest neighbor algorithm; YI, youden index; Sen,sensitivity; Spe, specificity.

#### 3.4.2 Constructing and evaluating the predictive model based on rGFR <60 mL/min·1.73 m^2^

**FIGURE 6:**
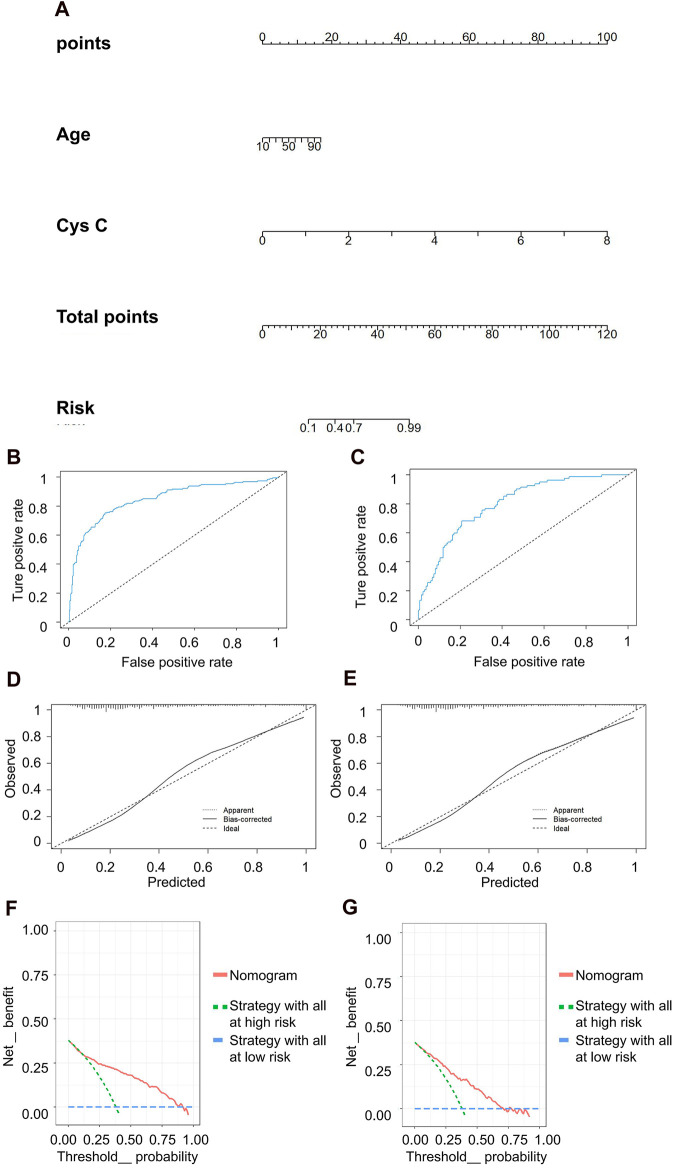
Clinical efficacy analysis for LR model based on rGFR < 60 mL/min 1.73 m^2^. **(A)** Nomogram prediction model. **(B,C)** ROC curves for training set and testing set. **(D,E)** Calibration outcome on training set and testing set. **(F,G)** DCA for training set and testing set. LR, logistic regression; rGFR, reference glomerular filtration rate; ROC, receiver operating characteristic; DCA, decision curve analysis.

In the discrimination of moderate kidney injury, age and Cys C were utilized as independent variables, with rGFR at 60 mL/min 1.73 m^2^ serving as the dependent variable. The accuracy rates achieved by these models were as follows: LR −78.082%, NN −60.731%, SVM −60.731%, NB −66.667%, and KNN −63.927%. Concurrently, LR maintained the highest AUC value at 0.834 (Table 5). The nomogram depicted Cys C as the most significant contributor to the prediction model (Figure 6A). The linear predictor for an individual LR model was established as: −6.030 + (0.039 × age) + (2.624 × Cys C). The model’s classification ability was displayed in Figures 6B,C. In the final LR model, there were the AUC, accuracy, recall rate, F1 score of training set and testing set (0.846 vs 0.800, 0.797 vs 0.733, 0.747 vs 0.683, 0.736 vs 0.659, respectively). The calibration curve closely mirrored the 45° line, and the DCA curve predominantly favored the upper right side of the two decision curves.

## 4 Discussion

In recent years, several equations have been developed to estimate glomerular filtration rate (eGFR), but their practicality varies. A study conducted by Jeong TD et al. showed that the EKFC equation exhibited the least bias and the highest P30, whereas the CKD-EPI_2009_ equation displayed lower bias compared to the CKD-EPI_2021_ equation (Jeong et al., 2023). Liefu Ye et al. (Li et al., 2022) observed the concordance and accuracy of 14 eGFR equations with rGFR in 50 renal tumor patients in China, suggesting that an eGFR equation constructed using both Scr and CysC might not necessarily outperform an equation constructed using only one of them. Among all the equations tested, FAS_Scr-SCysC_ performed the best in assessing postoperative GFR in renal tumor patients. Our research results indicate that there was inadequate agreement among the equations when applied to healthy adults. Nevertheless, the CG formula, CKD-EPI_ASR-Scr_, and CKD-EPI_2021-Scr_ demonstrated higher concordances with rGFR compared to other eGFR equations. Furthermore, we identified factors influencing rGFR and developed a machine learning logistic regression model, significantly enhancing our ability to distinguish renal function damage.

Participants were initially recruited from the health examination center as a healthy population. The BMI results indicated that the median BMI of the study population was in the overweight range, closely aligning with the 2023 obesity survey results of the adult population in China, which was conducted across 519 health check-up centers in 243 cities (Chen et al., 2023). The evaluation values obtained from each eGFR equation were consistently higher than 110 mL/min 1.73 m^2^, predominantly falling within the G1 phase, signifying that kidney function was close to normal levels. It is noteworthy that only CKD-EPI_ASR-Scr_-CKD-EPI_2021-Scr_ demonstrated a concordance > 0.9, possibly because the latter evolved from the former. These findings remained consistent across different subgroups categorized by sex, age, and BMI.

To gain a deeper understanding of these equations’ ability to reflect renal function, we collected data from patients visiting the Department of Nuclear Medicine and observed the concordance between eGFRs and rGFR. From a comprehensive population perspective, the CG formula, CKD-EPI_ASR-Scr_ and CKD-EPI_2021-Scr_ exhibited stronger correlations and concordance with rGFR, particularly CKD-EPI_ASR-Scr_. Peng Xie et al. (Wang et al., 2016) revealed that the CKD-EPI formula customized for Asians provides more precise GFR estimation in Chinese CKD patients in general practice. This precision was especially evident in the higher GFR group, underscoring the suitability of the CKD-EPI formula for the Chinese population. Interesting, the concordance between the CG formula and rGFR was highest in each subgroup based on gender, age, as well as in subgroups with BMI < 28 kg/m^2^. However, in the obese population, CKD-EPI_ASR-Scr_ demonstrated the best concordance with rGFR. Consequently, the CG formula equation was preferred for assessing GFR in populations with BMI < 28 kg/m^2^, while the CKD-EPI_ASR-Scr_ equation was favored in obese populations.

The existing literature primarily focuses on assessing the accuracy of each equation in estimating true renal function. However, in clinical practice, medical decisions often hinge on the medical staff’s ability to gauge the extent of kidney damage. Therefore, the equation’s capacity to accurately determine the actual extent of kidney damage may be of greater significance. It is noteworthy that the ROC curve analysis results revealed limited discriminatory power of each equation in distinguishing between different stages of renal impairment. This limitation may stem from the prevalent overestimation of GFR in all 10 eGFR equations utilized. This issue has not received significant attention in the past but warrants clinical resolution.

Advancements in diagnostic technology can lead to more effective diagnostic strategies based on existing indicators, rather than solely pursuing new, higher-value indicators. The development of risk prediction models using machine learning is a crucial step in enhancing the accuracy and efficiency of diagnosis and prognosis assessment in the era of big data. In 2022, the validation of the international IgA nephropathy risk prediction tool was performed through a study in Norway involving 306 IgA nephropathy patients diagnosed by renal biopsy pathology (with a median follow-up of 17.1 years). The results indicated that the international IgAN prediction tool sometimes outperformed the clinical decision support system for IgA nephropathy (Haaskjold et al., 2023). These prediction models can effectively assess the risk of kidney injury following various types of organ damage (Ravizza et al., 2019; Bjork et al., 2020; Wu et al., 2023), and predict the risk of functional impairment in other organs following kidney damage (Meyer et al., 2018). In addition, the risk of chronic kidney progression after acute kidney injury can also be predicted by building a prediction model (James et al., 2017). Moreover, a wide range of algorithms is continually emerging. LR is a classic probability statistical classification model that can quantify the degree of influence of the independent variable on the dependent variable and had been widely used in binary dependent variable modeling (Liu et al., 2022). Other models include SVM, decision trees, random forests, NN, NB, among others (Xue et al., 2021; Neyra et al., 2023).

In this experiment, the population was categorized into two groups based on rGFR levels: rGFR < 90 or < 60 mL/min 1.73 m^2^, in order to identify the primary influencing factors. Regardless of whether the grouping was based on rGFR < 60 or < 90 mL/min 1.73 m^2^, age, SBP, Cys C, and Scr exhibited statistical differences between the groups and strong correlations with rGFR. After automatically eliminating Scr using a regression model, the results of stepwise regression analysis revealed that height had a significant positive impact on rGFR, while age, height, SBP, and Cys C had a significant negative impact on rGFR, consistent with the findings of LASSO regression analysis. When it came to identifying rGFR < 90 mL/min 1.73 m^2^, the ROC curve results indicated that age, SBP, and Cys C had diagnostic value, while only age and Cys C remained relevant when identifying rGFR < 60 mL/min 1.73 m^2^. The nomogram illustrated that Cys C made the most significant contribution to diagnosing kidney damage.

Subsequently, we constructed five prediction models, including LR, NN, SVM, NB, and KNN. Random forest and decision tree were not employed in this study due to their reliance on clinical features. The test results demonstrated that LR exhibited the best accuracy and predictive ability among the models. In terms of identifying mild or moderate renal injury, the AUC of the LR model significantly outperformed that of individual indicators and previous eGFR equations. Furthermore, the results of the calibration curve and DCA indicated that the LR model possessed strong clinical applicability. This model effectively identified kidney damage without incorporating Scr (Pei et al., 2013; Inker et al., 2021), reinforcing the notion that Cys C is no less capable of reflecting kidney function than Scr. However, it is important to highlight that the construction process of the LR model utilized a limited number of indicators. Thus, expanding the range of screening indicators may be necessary to develop a more robust LR model and enhance the diagnostic efficiency for chronic kidney disease.

## 5 Conclusion

In conclusion, our findings highlight that the concordance of CKD-EPI_ASR-Scr_ and CKD-EPI_2021-Scr_ was the highest in the population of central China. Moreover, the CG formula, CKD-EPI_ASR-Scr_, and CKD-EPI_2021-Scr_ proved to be more accurate in reflecting true kidney function but exhibited limitations in the staging diagnosis of CKD. More importantly, the diagnostic prediction model learning demonstrated strong clinical applicability in identifying mild or moderate kidney injury. These results lay a valuable foundation for future studies aimed at assessing renal function and developing predictive equations.

## Data Availability

The original contributions presented in the study are included in the article/Supplementary Material, further inquiries can be directed to the corresponding author.

## Abbreviations

eGFR, estimating glomerular filtration rate; rGFR, referenced glomerular filtration rate; CKD, chronic kidney disease; CG, Cockcroft-Gault; MDRD, Modification of Diet in Renal Disease Research Group; CKD-EPI, Chronic Kidney Disease Epidemiology Collaboration equation; FAS, Full Age Spectrum; EKFC, European Kidney Function Consortium; EFLM, European Federation of Experimental Medicine; LR, logistic regression; ML, machine learning; NN, neural network; SVM, support vector machines; NB, naive Bayes; KNN, k-nearest neighbor algorithm; ROC, receiver operating characteristic; DCA, decision curve analysis; ICC, intraclass correlation coefficient; LASSO, least absolute shrinkage and selection operator.
