# Supplementary material for: Evaluation of the clinical value of 10 estimating glomerular filtration rate equations and construction of a prediction model for kidney damage in adults from central China
Source: Front Mol Biosci. 2024 Jun 13;11:1408503. doi: 10.3389/fmolb.2024.1408503 (PMC11208320; doi:10.3389/fmolb.2024.1408503)
Supplement: Supplementary file 1 [file Table1.DOC]

| Equation | Name |  | Equation |
| --- | --- | --- | --- |
| eGFR1 | CG formula |  | (140-Age)×Weight/(72/Scr)×(0.85 if female) |
| eGFR2 | MDRD Ⅰ (simplified) |  | 186×Scr^-1.154^×Age^-0.203^×0.742 (if female) |
| eGFR3 | MDRD Ⅱ (simplified) |  | 175×Scr^-1.154^×Age^-0.203^×0.742 (if female) ×1.212 (if black) |
| eGFR4 | MDRD Ⅲ (Chinese) |  | 186×SCr^-1.154^×Age^-0.203^×0.742 (if female) ×1.233 (if Chinese) |
| eGFR5 | CKD-EPI_2009-Scr_ | Male (Scr≤0.9)  Male (Scr＞0.9)  Female (Scr≤0.7)  Female (Scr＞0.7) | 141×(Scr/0.9) ^−0.411^×0.993^Age^  141×(Scr/0.9) ^−1.209^×0.993^Age^  144×(Scr/0.7) ^−0.329^×0.993^Age^  144×(Scr/0.7) ^−1.209^×0.993^Age^ |
| eGFR6 | FAS | 2≤Age≤40  Age＞40 | 107.3/(Scr/QScr)  0.988 ^(Age−40)^ × 107.3/(Scr/QScr)  QScr 0.7 mg/dL (if female); QScr 0.9 mg/dL (if male) |
| eGFR7 | BIS |  | 3736 × Scr^−0.87^ × Age^−0.95^ × 0.82 (if female) |
| eGFR8 | EKFC | Male (Scr/0.9＜1)  Male (Scr/0.9≥1)  Female (Scr/0.7＜1)  Female (Scr/0.7≥1) | 107.3×(Scr/0.9)^-0.322^×0.990^(Age-40)^  107.3×(Scr/0.9)^-1.132^×0.990^(Age-40)^  107.3×(Scr/0.7)^-0.322^×0.990^(Age-40)^  107.3×(Scr/0.7)^-1.132^×0.990^(Age-40)^ |
| eGFR9 | CKD-EPI_ASR-Scr_ | Male (Scr≤0.9)  Male (Scr＞0.9)  Female (Scr≤0.7)  Female (Scr＞0.7) | 149×(Scr/0.9)^-0.415^×0.993^Age^  149×(Scr/0.9)^-1.210^×0.993^Age^  151×(Scr/0.7)^-0.328^×0.993^Age^  151×(Scr/0.7)^-1.210^×0.993^Age^ |
| eGFR10 | CKD-EPI_2021-Scr_ | Male (Scr≤0.9)  Male (Scr＞0.9)  Female (Scr≤0.7)  Female (Scr＞0.7) | 142×(Scr/0.9)^-0.302^×0.9938^Age^  142×(Scr/0.9)^-1.200^×0.9938^Age^  142×(Scr/0.7)^-0.241^×0.9938^Age^  142×(Scr/0.7)^-1.200^×0.9938^Age^ |

Table S1 10 equations based on serum creatinine used to calculate eGFR.

Scr, serum creatinine; eGFR, estimated glomerular filtration rate; CG, Cockcroft-Gault; MDRD, Modification of Diet in Renal Disease; CKD-EPI, Chronic Kidney Disease Epidemiological Collaboration; FAS, Full Age Spectrum; BIS, Berlin Initiative Study; EKFC, European Kidney Function Consortium.

Table S2 Distribution across eGFR categories in the total participants

| Equation | G1, n (%) | G2, n (%) | G3, n (%) | G3b, n (%) | G4, n (%) | G5, n (%) |
| --- | --- | --- | --- | --- | --- | --- |
| eGFR1 | 65460(86.62) | 9300(12.31) | 671(0.89) | 109(0.14) | 20(0.03) | 13(0.02) |
| eGFR2 | 63158(83.57) | 11870(15.71) | 642(0.85) | 60(0.08) | 26(0.03) | 17(0.02) |
| eGFR3 | 59223(78.37) | 15355(20.32) | 858(1.14) | 96(0.13) | 24(0.03) | 17(0.02) |
| eGFR4 | 72377(95.77) | 3061(4.05) | 72(0.10) | 36(0.05) | 13(0.02) | 14(0.02) |
| eGFR5 | 68022(90.01) | 7307(9.67) | 195(0.26) | 28(0.04) | 15(0.02) | 6(0.01) |
| eGFR6 | 70182(92.87) | 5268(6.97) | 73(0.10) | 26(0.03) | 12(0.02) | 12(0.02) |
| eGFR7 | 64723(85.64) | 9963(13.18) | 805(1.07) | 61(0.08) | 16(0.02) | 5(0.01) |
| eGFR8 | 61261(81.06) | 13847(18.32) | 420(0.56) | 26(0.03) | 15(0.02) | 4(0.01) |
| eGFR9 | 67961(89.93) | 7276(9.63) | 244(0.32) | 52(0.07) | 24(0.03) | 16(0.02) |
| eGFR10 | 67625(89.48) | 7617(10.08) | 241(0.32) | 50(0.07) | 24(0.03) | 16(0.02) |

eGFR, estimated glomerular filtration rate.

Table S3 Concordance among equations in the staging of CKD

| Equation | eGFR1 | eGFR2 | eGFR3 | eGFR4 | eGFR5 | eGFR6 | eGFR7 | eGFR8 | eGFR9 | eGFR10 |
| --- | --- | --- | --- | --- | --- | --- | --- | --- | --- | --- |
| eGFR1 | 1.000 |  |  |  |  |  |  |  |  |  |
| eGFR2 | 0.269(0.26-0.278) | 1.000 |  |  |  |  |  |  |  |  |
| eGFR3 | 0.270(0.262-0.278) | 0.424(0.416-0.432) | 1.000 |  |  |  |  |  |  |  |
| eGFR4 | 0.221(0.210-0.231) | 0.375(0.365-0.385) | 0.242(0.234-0.25) | 1.000 |  |  |  |  |  |  |
| eGFR5 | 0.485(0.476-0.494) | 0.329(0.32-0.338) | 0.307(0.299-0.315) | 0.268(0.257-0.28) | 1.000 |  |  |  |  |  |
| eGFR6 | 0.192(0.182-0.202) | 0.195(0.186-0.204) | 0.176(0.168-0.184) | 0.225(0.212-0.239) | 0.343(0.332-0.354) | 1.000 |  |  |  |  |
| eGFR7 | 0.587(0.579-0.595) | 0.328(0.319-0.336) | 0.338(0.33-0.346) | 0.236(0.226-0.246) | 0.604(0.596-0.612) | 0.195(0.185-0.204) | 1.000 |  |  |  |
| eGFR8 | 0.446(0.438-0.454) | 0.216(0.208-0.225) | 0.221(0.213-0.229) | 0.133(0.125-0.141) | 0.58(0.572-0.588) | 0.154(0.146-0.162) | 0.600(0.592-0.607) | 1.000 |  |  |
| eGFR9 | 0.365(0.356-0.375) | 0.664(0.656-0.671) | 0.348(0.34-0.357) | 0.572(0.561-0.583) | 0.444(0.434-0.455) | 0.216(0.205-0.227) | 0.427(0.418-0.436) | 0.287(0.279-0.296) | 1.000 |  |
| eGFR10 | 0.358(0.349-0.368) | 0.683(0.675-0.691) | 0.351(0.343-0.36) | 0.554(0.543-0.565) | 0.441(0.431-0.452) | 0.221(0.21-0.233) | 0.413(0.403-0.422) | 0.281(0.272-0.289) | 0.964(0.961-0.967) | 1.000 |

eGFR, estimated glomerular filtration rate.

Table S4 eGFR distribution in sexual groups

| Characteristic | Female (n=32542) | Male (n=43031) | *p*-Value |
| --- | --- | --- | --- |
| Age (years) | 43.00 (32.00,54.00) | 44.00 (32.00, 55.00) | 0.000 |
| BMI (kg/m^2^) | 24.07 (21.73,26.49) | 24.09 (21.76,26.49) | 0.003 |
| Scr (mg/dL) | 0.67(0.56, 0.79) | 0.67(0.56, 0.78) | 0.180 |
| eGFR (mL/min/1.73 m^2^) |  |  |  |
| eGFR1 | 131.27 (108.36, 157.29) | 130.42 (107.19, 157.26) | 0.000 |
| eGFR2 | 90.85 (111.51, 135.75) | 150.22 (122.44, 183.30) | 0.000 |
| eGFR3 | 124.17 (100.35, 154.54) | 124.21 (100.12, 154.76) | 0.053 |
| eGFR4 | 137.49 (112.01, 167.38) | 185.22 (150.96, 226.00) | 0.000 |
| eGFR5 | 118.53 (105.59, 132.25) | 118.13 (105.04, 131.97) | 0.000 |
| eGFR6 | 135.50 (113.69, 164.49) | 136.15 (113.82, 164.49) | 0.000 |
| eGFR7 | 148.51 (113.11, 197.21) | 146.94 (111.27, 197.36) | 0.000 |
| eGFR8 | 111.46 (96.83, 127.54) | 110.94 (96.19, 127.54) | 0.000 |
| eGFR9 | 108.82 (94.56,122.02) | 123.23 (110.80,135.61) | 0.000 |
| eGFR10 | 106.68(93.66,118.05) | 117.76 (107.71,127.41) | 0.000 |

BMI, body mass index (calculated as weight/height^2^); Scr, serum creatinine; eGFR, estimated glomerular filtration rate.

Table S5 Distribution across eGFR categories in sexual groups

| Stages | Sex | eGFR1 | eGFR2 | eGFR3 | eGFR4 | eGFR5 | eGFR6 | eGFR7 | eGFR8 | eGFR9 | eGFR10 | |
| --- | --- | --- | --- | --- | --- | --- | --- | --- | --- | --- | --- | --- |
| G1, n (%) | female | 28389  (87.24) | 21835  (67.10) | 25535  (78.47) | 29604  (90.97) | 29422  (90.41) | 30171  (92.71) | 28121  (86.41) | 26659  (81.925) | 26317  (80.87) | 26021  (79.96) |  |
|  | male | 37071  (86.15) | 41323  (96.03) | 33688  (78.29) | 42773  (99.40) | 38600  (89.70) | 40011  (92.98) | 36602  (85.06) | 34602  (80.41) | 41644  (96.78) | 41604  (96.68) |  |
| G2, n (%) | female | 3870  (11.89) | 10260  (31.53) | 6578  (20.21) | 2844  (8.74) | 3000  (9.22) | 2314  (7.11) | 4088  (12.56) | 5691  (17.49) | 5969  (18.34） | 6270  (19.27) |  |
|  | male | 5430  (12.62) | 1610  (3.74) | 8777  (20.40) | 217  (0.50) | 4307  (10.01) | 2954  (6.86) | 5875  (13.65) | 8156  (18.95) | 1307  (3.04) | 1347  (3.13) |  |
| G3, n (%) | female | 224  (0.69) | 380  (1.17) | 370  (1.14) | 52  (0.16) | 99  (0.30) | 32  (0.10) | 299  (0.92) | 173  (0.53) | 196  (0.60) | 192  (0.59) |  |
|  | male | 447  (1.04) | 62  (0.14) | 488  (1.13) | 20  (0.05) | 96  (0.22) | 41  (0.10) | 506  (1.18) | 247  (0.57) | 48  (0.11) | 49  (0.11) |  |
| G3b, n (%) | female | 41  (0.13) | 40  (0.13) | 38  (0.12) | 27  (0.08) | 11  (0.03) | 10  (0.03) | 26  (0.08) | 9  (0.03) | 35  (0.11) | 35  (0.11) |  |
|  | male | 68  (0.16) | 20  (0.05) | 58  (0.13) | 9  (0.02) | 17  (0.04) | 16  (0.04) | 35  (0.08) | 17  (0.04) | 17  (0.04) | 15  (0.03) |  |
| G4, n (%) | female | 13  (0.04) | 20  (0.06) | 15  (0.05) | 9  (0.03) | 8  (0.02) | 9  (0.03) | 6(0.02) | 9  (0.03) | 19  (0.06) | 18  (0.06) |  |
|  | male | 7  (0.02) | 6  (0.01) | 9  (0.02) | 4  (0.01) | 7  (0.02) | 3  (0.01) | 10  (0.02) | 6  (0.01) | 1  (0.00) | 10  (0.02) |  |
| G5, n (%) | female | 5  (0.02) | 7  (0.02) | 6(0.018) | 6  (0.02) | 2  (0.01) | 6  (0.02) | 2  (0.01) | 1  (0.00) | 6  (0.02) | 6  (0.02) |  |
|  | male | 8  (0.02) | 10  (0.02) | 11  (0.03) | 8  (0.02) | 4  (0.01) | 6  (0.01) | 3  (0.01) | 3  (0.01) | 5  (0.01) | 6  (0.01) |  |

eGFR, estimated glomerular filtration rate.

Table S6 Concordance among equations in female group

| Equation | eGFR1 | eGFR2 | eGFR3 | eGFR4 | eGFR5 | eGFR6 | eGFR7 | eGFR8 | eGFR9 | eGFR10 |
| --- | --- | --- | --- | --- | --- | --- | --- | --- | --- | --- |
| eGFR1 | 1.000 |  |  |  |  |  |  |  |  |  |
| eGFR2 | 0.273(0.263-0.284) | 1.000 |  |  |  |  |  |  |  |  |
| eGFR3 | 0.271(0.258-0.284) | 0.547(0.537-0.557) | 1.000 |  |  |  |  |  |  |  |
| eGFR4 | 0.364(0.348-0.38) | 0.343(0.333-0.353) | 0.447(0.435-0.46) | 1.000 |  |  |  |  |  |  |
| eGFR5 | 0.482(0.467-0.496) | 0.335(0.325-0.345) | 0.299(0.287-0.311) | 0.413(0.397-0.429) | 1.000 |  |  |  |  |  |
| eGFR6 | 0.202(0.186-0.218) | 0.178(0.169-0.187) | 0.178(0.165-0.19) | 0.302(0.284-0.321) | 0.355(0.338-0.372) | 1.000 |  |  |  |  |
| eGFR7 | 0.57(0.558-0.583) | 0.371(0.361-0.381) | 0.335(0.323-0.348) | 0.41(0.396-0.425) | 0.599(0.587-0.612) | 0.202(0.187-0.216) | 1.000 |  |  |  |
| eGFR8 | 0.428(0.415-0.44) | 0.268(0.257-0.278) | 0.217(0.205-0.23) | 0.243(0.23-0.257) | 0.581(0.569-0.594) | 0.159(0.146-0.172) | 0.600(0.592-0.607) | 1.000 |  |  |
| eGFR9 | 0.421(0.409-0.434) | 0.631(0.622-0.64) | 0.496(0.485-0.508) | 0.593(0.581-0.605) | 0.483(0.47-0.496) | 0.234(0.221-0.248) | 0.427(0.418-0.436) | 0.38(0.368-0.393) | 1.000 |  |
| eGFR10 | 0.406(0.394-0.419) | 0.655(0.646-0.664) | 0.501(0.489-0.512) | 0.571(0.559-0.583) | 0.477(0.464-0.489) | 0.241(0.227-0.254) | 0.413(0.403-0.422) | 0.366(0.354-0.379) | 0.961(0.958-0.965) | 1.000 |

eGFR, estimated glomerular filtration rate.

Table S7 Concordance among equations in male group

| Equation | eGFR1 | eGFR2 | eGFR3 | eGFR4 | eGFR5 | eGFR6 | eGFR7 | eGFR8 | eGFR9 | eGFR10 |
| --- | --- | --- | --- | --- | --- | --- | --- | --- | --- | --- |
| eGFR1 | 1.000 |  |  |  |  |  |  |  |  |  |
| eGFR2 | 0.272(0.258-0.286) | 1.000 |  |  |  |  |  |  |  |  |
| eGFR3 | 0.269(0.258-0.28) | 0.269(0.258-0.279) | 1.000 |  |  |  |  |  |  |  |
| eGFR4 | 0.081(0.069-0.093) | 0.301(0.27-0.333) | 0.055(0.047-0.063) | 1.000 |  |  |  |  |  |  |
| eGFR5 | 0.488(0.476-0.5) | 0.326(0.311-0.342) | 0.313(0.302-0.324) | 0.1(0.086-0.113) | 1.000 |  |  |  |  |  |
| eGFR6 | 0.185(0.173-0.197) | 0.232(0.214-0.25) | 0.175(0.165-0.186) | 0.108(0.09-0.126) | 0.335(0.320-0.349) | 1.000 |  |  |  |  |
| eGFR7 | 0.598(0.588-0.608) | 0.271(0.258-0.284) | 0.34(0.33-0.351) | 0.071(0.06-0.081) | 0.608(0.597-0.618) | 0.19(0.178-0.201) | 1.000 |  |  |  |
| eGFR8 | 0.459(0.448-0.469) | 0.159(0.149-0.17) | 0.224(0.213-0.234) | 0.038(0.031-0.044) | 0.578(0.568-0.589) | 0.15(0.14-0.161) | 0.608(0.599-0.618) | 1.000 |  |  |
| eGFR9 | 0.302(0.288-0.316) | 0.626(0.605-0.648) | 0.194(0.184-0.205) | 0.359(0.324-0.394) | 0.391(0.376-0.407) | 0.18(0.162-0.198) | 0.289(0.277-0.302) | 0.194(0.184-0.205) | 1.000 |  |
| eGFR10 | 0.304(0.29-0.318) | 0.629(0.608-0.65) | 0.194(0.183-0.205) | 0.351(0.316-0.386) | 0.392(0.376-0.408) | 0.183(0.165-0.201) | 0.29(0.277-0.302) | 0.195(0.184-0.205) | 0.961(0.953-0.968) | 1.000 |

eGFR, estimated glomerular filtration rate.

Table S7 Distribution across eGFR categories in different age groups

| Stage | Age (years) | eGFR1 | eGFR2 | eGFR3 | eGFR4 | eGFR5 | eGFR6 | eGFR7 | eGFR8 | eGFR9 | eGFR10 |
| --- | --- | --- | --- | --- | --- | --- | --- | --- | --- | --- | --- |
| G1, n (%) | 18-39 | 29247  (97.93) | 26598  (89.06) | 25433  (85.16) | 29298  (98.10) | 29029  (97.20) | 27791  (93.06) | 29811  (99.82) | 29169  (97.67) | 28727  (96.19) | 28504  (95.45) |
|  | 40-60 | 29234  (88.71) | 26851  (81.48) | 25042  (75.99) | 31304  (95.00) | 30374  (92.17) | 30619  (92.92) | 29096  (88.30) | 27628  (83.84) | 29614  (89.87) | 29638  (89.94) |
|  | ＞60 | 6979  (54.71) | 9709  (76.11) | 8748  (68.58) | 11795  (92.47) | 8619  (67.57) | 11772  (92.29) | 5816  (45.59) | 4464  (35.00) | 9761  (76.52) | 9802  (76.84) |
| G2, n (%) | 18-39 | 587  (1.97) | 3173  (10.62) | 4254  (14.24) | 559  (1.87) | 808  (2.71) | 2032  (6.80) | 43  (0.14) | 675  (2.264) | 1090  (3.65) | 1308  (4.38) |
|  | 40-60 | 3595  (10.91) | 5820  (17.66) | 7454  (22.62) | 1569  (4.76) | 2488  (7.55) | 2269  (6.89) | 3773  (11.45) | 5224  (15.85) | 3154  (9.57) | 3143  (9.54) |
|  | ＞60 | 5118  (40.12) | 2877  (22.55) | 3647  (28.59) | 933  (7.31) | 4011  (31.44) | 967  (7.58) | 6147  (48.19) | 7948  (62.31) | 2848  (22.33) | 2820  (22.11) |
| G3, n (%) | 18-39 | 17  (0.06) | 68  (0.22) | 156  (0.52) | 11  (0.04) | 16  (0.05) | 23  (0.08) | 2  (0.01) | 16  (0.05) | 28  (0.09) | 33  (0.11) |
|  | 40-60 | 85  (0.26) | 218  (0.66) | 372  (1.13) | 41  (0.12) | 63  (0.19) | 40  (0.12) | 53  (0.16) | 77  (0.23) | 150  (0.46) | 139  (0.42) |
|  | ＞60 | 569  (4.46) | 156  (1.22) | 330  (2.59) | 20  (0.16) | 116  (0.91) | 10  (0.08) | 750  (5.88) | 327  (2.56) | 130  (1.02) | 119  (0.93) |
| G3b, n (%) | 18-39 | 3(0.01) | 12  (0.04) | 10  (0.03) | 6  (0.02) | 8  (0.03) | 8  (0.03) | 1  (0.00) | 1  (0.00) | 8  (0.03) | 7  (0.02) |
|  | 40-60 | 25  (0.08) | 40  (0.12) | 60  (0.18) | 24  (0.07) | 14  (0.04) | 14  (0.04) | 21  (0.06) | 12  (0.04) | 22  (0.07) | 20  (0.06) |
|  | ＞60 | 81  (0.63) | 8  (0.06) | 26  (0.20) | 6  (0.05) | 6  (0.05) | 4  (0.03) | 39  (0.31) | 13  (0.10) | 11  (0.09) | 9  (0.07) |
| G4, n (%) | 18-39 | 5  (0.02) | 5  (0.02) | 4  (0.01) | 3  (0.01) | 1  (0.00) | 4  (0.01) | 7  (0.02) | 2  (0.01) | 4  (0.01) | 5  (0.02) |
|  | 40-60 | 8  (0.02) | 17  (0.05) | 17  (0.05) | 9  (0.03) | 12  (0.04) | 7  (0.02) | 7  (0.02) | 11  (0.03) | 8  (0.02) | 8  (0.02) |
|  | ＞60 | 7  (0.05) | 4  (0.03) | 3  (0.02) | 1  (0.01) | 2  (0.02) | 1  (0.01) | 2  (0.02) | 2  (0.02) | 4  (0.03) | 4  (0.03) |
| G5, n (%) | 18-39 | 5  (0.02) | 8  (0.03) | 7  (0.02) | 7  (0.02) | 2  (0.01) | 6  (0.02) | 0  (0.00) | 1  (0.00) | 7  (0.02) | 7  (0.02) |
|  | 40-60 | 6  (0.02) | 7  (0.02) | 8  (0.02) | 6  (0.02) | 2  (0.01) | 4  (0.01) | 3  (0.01) | 1  (0.00) | 2  (0.01) | 5  (0.02) |
|  | ＞60 | 2  (0.02) | 2  (0.02) | 2  (0.02) | 1  (0.01) | 2  (0.02) | 2  (0.02) | 2  (0.02) | 2  (0.02) | 2  (0.02) | 2  (0.02) |

eGFR, estimated glomerular filtration rate.

Table S8 The baseline data of participants in different age groups

| Characteristic | Adults aged 18-39 years  (n=29864) | Adults aged 40-60 years  (n=32953) | Adults aged ＞60 years  (n=12756) | *p*-Value |
| --- | --- | --- | --- | --- |
| Sex (male/female) | 16840/13024 | 18570/14383 | 7621/5135^***###^ | 0.000 |
| BMI (kg/m^2^) | 24.08 (21.77, 26.49) | 24.08 (21.73, 26.49) ^***^ | 24.11 (21.74, 26.49) ^###^ | 0.000 |
| Scr (mg/dL) | 0.56(0.67, 0.78) | 0.55(0.66, 0.78) ^***^ | 0.56(0.67, 0.78) ^###^ | 0.000 |
| eGFR (mL/min/1.73 m^2^) |  |  |  |  |
| eGFR1 | 150.26(128.99, 175.79) | 123.51(105.62, 145.13) ^***^ | 96.21(80.68, 114.03) ^***###^ | 0.000 |
| eGFR2 | 143.90(117.33, 180.82) | 129.94(105.78, 161.92) ^***^ | 122.45(99.75, 151.71) ^***###^ | 0.000 |
| eGFR3 | 133.12(107.97, 165.60) | 120.00(97.58, 148.99) ^***^ | 111.97(90.83, 139.46) ^***###^ | 0.000 |
| eGFR4 | 177.42(144.67, 222.95) | 160.22(130.43, 199.65) ^***^ | 150.976513(122.99, 187.05) ^***###^ | 0.000 |
| eGFR5 | 129.24(118.86, 141.91) | 113.28(103.88, 124.38) ^***^ | 98.59(89.89, 109.07) ^***###^ | 0.000 |
| eGFR6 | 136.90(114.68, 165.44) | 135.50(113.37, 163.85) ^***^ | 134.23(112.35, 163.54) ^***^ | 0.000 |
| eGFR7 | 204.35(168.79, 249.27) | 128.09(107.45, 151.93) ^***^ | 92.55(77.82, 110.35) ^***###^ | 0.000 |
| eGFR8 | 127.07(114.75, 139.26) | 105.23(95.05, 115.43) ^***^ | 86.53(77.17, 96.01) ^***###^ | 0.000 |
| eGFR9 | 130.10 (120.72,140.37) | 113.66 (105.23,122.75) ^***^ | 99.36(90.90,107.45) ^***###^ | 0.000 |
| eGFR10 | 124.53(117.44,131.79) | 110.51(103.98,117.03) ^***^ | 98.11 (90.94,104.05) ^***###^ | 0.000 |

BMI, body mass index (calculated as weight/height^2^ ); Scr, serum creatinine; eGFR, estimated glomerular filtration rate. vs. adults aged 18-39 years group, ^***^*p*＜0.001; vs. Adults aged 40-60 years group, ^###^*p*＜0.001.

Table S9 Concordance among equations in 18-39 years group

| Equation | eGFR1 | eGFR2 | eGFR3 | eGFR4 | eGFR5 | eGFR6 | eGFR7 | eGFR8 | eGFR9 | eGFR10 |
| --- | --- | --- | --- | --- | --- | --- | --- | --- | --- | --- |
| eGFR1 | 1.000 |  |  |  |  |  |  |  |  |  |
| eGFR2 | 0.167(0.147-0.186) | 1.000 |  |  |  |  |  |  |  |  |
| eGFR3 | 0.138(0.122-0.153) | 0.388(0.373-0.404) | 1.000 |  |  |  |  |  |  |  |
| eGFR4 | 0.324(0.281-0.367) | 0.297(0.276-0.317) | 0.187(0.171-0.204) | 1.000 |  |  |  |  |  |  |
| eGFR5 | 0.308(0.275-0.341) | 0.193(0.176-0.211) | 0.158(0.144-0.173) | 0.252(0.218-0.285) | 1.000 |  |  |  |  |  |
| eGFR6 | 0.213(0.189-0.238) | 0.246(0.228-0.264) | 0.207(0.192-0.223) | 0.168(0.144-0.191) | 0.391(0.368-0.414) | 1.000 |  |  |  |  |
| eGFR7 | 0.199(0.146-0.253) | 0.042(0.028-0.055) | 0.03(0.02-0.04) | 0.199(0.144-0.254) | 0.119(0.082-0.156) | 0.044(0.028-0.061) | 1.000 |  |  |  |
| eGFR8 | 0.297(0.263-0.332) | 0.156(0.139-0.173) | 0.134(0.12-0.147) | 0.251(0.216-0.285) | 0.843(0.824-0.862) | 0.329(0.306-0.353) | 0.095(0.059-0.13) | 1.000 |  |  |
| eGFR9 | 0.299(0.265-0.333) | 0.501(0.482-0.52) | 0.244(0.227-0.261) | 0.684(0.659-0.71) | 0.29(0.262-0.318) | 0.227(0.204-0.25) | 0.116(0.082-0.15) | 0.295(0.265-0.324) | 1.000 |  |
| eGFR10 | 0.282(0.251-0.313) | 0.571(0.553-0.589) | 0.266(0.249-0.282) | 0.606(0.58-0.633) | 0.308(0.281-0.335) | 0.251(0.229-0.274) | 0.099(0.07-0.129) | 0.294(0.267-0.322) | 0.911(0.899-0.922) | 1.000 |

eGFR, estimated glomerular filtration rate.

Table S10 Concordance among equations in 40-60 years group

| Equation | eGFR1 | eGFR2 | eGFR3 | eGFR4 | eGFR5 | eGFR6 | eGFR7 | eGFR8 | eGFR9 | eGFR10 |
| --- | --- | --- | --- | --- | --- | --- | --- | --- | --- | --- |
| eGFR1 | 1.000 |  |  |  |  |  |  |  |  |  |
| eGFR2 | 0.332(0.32-0.344) | 1.000 |  |  |  |  |  |  |  |  |
| eGFR3 | 0.291(0.28-0.302) | 0.37(0.359-0.382) | 1.000 |  |  |  |  |  |  |  |
| eGFR4 | 0.155(0.145-0.166) | 0.413(0.396-0.43) | 0.162(0.152-0.172) | 1.000 |  |  |  |  |  |  |
| eGFR5 | 0.495(0.485-0.506) | 0.454(0.441-0.467) | 0.374(0.362-0.385) | 0.24(0.226-0.253) | 1.000 |  |  |  |  |  |
| eGFR6 | 0.164(0.154-0.175) | 0.192(0.176-0.207) | 0.162(0.152-0.172) | 0.195(0.175-0.215) | 0.292(0.278-0.305) | 1.000 |  |  |  |  |
| eGFR7 | 0.582(0.573-0.592) | 0.409(0.399-0.42) | 0.403(0.392-0.413) | 0.161(0.152-0.17) | 0.613(0.604-0.622) | 0.095(0.087-0.103) | 1.000 |  |  |  |
| eGFR8 | 0.426(0.416-0.435) | 0.271(0.261-0.281) | 0.226(0.216-0.237) | 0.102(0.095-0.108) | 0.503(0.494-0.513) | 0.025(0.015-0.036) | 0.595(0.586-0.604) | 1.000 |  |  |
| eGFR9 | 0.371(0.359-0.382) | 0.74(0.728-0.752) | 0.31(0.298-0.321) | 0.465(0.447-0.483) | 0.508(0.495-0.521) | 0.176(0.16-0.192) | 0.416(0.406-0.426) | 0.286(0.276-0.295) | 1.000 |  |
| eGFR10 | 0.369(0.357-0.38) | 0.571(0.553-0.589) | 0.304(0.293-0.316) | 0.469(0.451-0.487) | 0.506(0.493-0.518) | 0.251(0.229-0.274) | 0.41(0.399-0.42) | 0.281(0.271-0.29) | 0.973(0.969-0.977) | 1.000 |

eGFR, estimated glomerular filtration rate.

Table S11 Concordance among equations in ＞60 years group

| Equation | eGFR1 | eGFR2 | eGFR3 | eGFR4 | eGFR5 | eGFR6 | eGFR7 | eGFR8 | eGFR9 | eGFR10 |
| --- | --- | --- | --- | --- | --- | --- | --- | --- | --- | --- |
| eGFR1 | 1.000 |  |  |  |  |  |  |  |  |  |
| eGFR2 | 0.227(0.212-0.241) | 1.000 |  |  |  |  |  |  |  |  |
| eGFR3 | 0.268(0.253-0.283) | 0.438(0.422-0.454) | 1.000 |  |  |  |  |  |  |  |
| eGFR4 | 0.12(0.11-0.13) | 0.408(0.389-0.426) | 0.253(0.238-0.268) | 1.000 |  |  |  |  |  |  |
| eGFR5 | 0.457(0.444-0.471) | 0.425(0.409-0.441) | 0.446(0.431-0.462) | 0.225(0.21-0.24) | 1.000 |  |  |  |  |  |
| eGFR6 | 0.109(0.1-0.119) | 0.16(0.142-0.177) | 0.157(0.143-0.171) | 0.262(0.233-0.29) | 0.198(0.184-0.213) | 1.000 |  |  |  |  |
| eGFR7 | 0.512(0.499-0.525) | 0.309(0.296-0.321) | 0.392(0.379-0.405) | 0.122(0.114-0.13) | 0.535(0.524-0.547) | 0.098(0.09-0.105) | 1.000 |  |  |  |
| eGFR8 | 0.347(0.333-0.361) | 0.186(0.175-0.197) | 0.231(0.219-0.244) | 0.064(0.058-0.07) | 0.367(0.355-0.38) | 0.029(0.022-0.035) | 0.547(0.533-0.56) | 1.000 |  |  |
| eGFR9 | 0.311(0.297-0.325) | 0.73(0.717-0.744) | 0.427(0.411-0.443) | 0.417(0.398-0.435) | 0.508(0.492-0.523) | 0.159(0.141-0.177) | 0.357(0.345-0.368) | 0.215(0.204-0.226) | 1.000 |  |
| eGFR10 | 0.308(0.294-0.322) | 0.71(0.696-0.725) | 0.414(0.397-0.43) | 0.424(0.405-0.443) | 0.505(0.489-0.521) | 0.159(0.141-0.176) | 0.348(0.336-0.359) | 0.209(0.199-0.22) | 0.968(0.964-0.973) | 1.000 |

eGFR, estimated glomerular filtration rate.

Table S12 The baseline data of participants in groups with different weight levels

| Characteristic | Underweight  (n=2547) | Normal weight  (n=32055) | Overweight  (n=29232) | Obesity  (n=11739) | *p*-Value |
| --- | --- | --- | --- | --- | --- |
| Sex (male/female) | 1444/1103 | 18225/13830 | 16674/12558 | 6688/5051 | 0.964 |
| Age (years) | 45 (33,56) | 45 (33,56) | 45 (33,56) | 45 (33,56) | 0.822 |
| Scr (mg/dL) | 0.59 (0.52, 0.68) | 0.62 (0.54, 0.74) ^***^ | 0.71 (0.59, 0.81) ^***###^ | 0.72 (0.61, 0.82) ^***###$$$^ | 0.000 |
| eGFR (mL/min/1.73 m^2^) |  |  |  |  |  |
| eGFR1 | 99.72 (83.30, 118.25) | 116.09 (96.72, 138.31) ^***^ | 128.63 (107.08, 152.71) ^***###^ | 149.11 (123.38, 177.95) ^***###$$$^ | 0.000 |
| eGFR2 | 140.34 (114.99, 169.52) | 129.87 (105.28, 160.45) ^***^ | 116.11 (94.55, 143.37) ^***###^ | 113.51 (93.25, 139.97) ^***###$$$^ | 0.000 |
| eGFR3 | 132.32 (109.36, 160.30) | 122.62 (99.24, 151.27) ^***^ | 109.26 (89.17, 134.71) ^***###^ | 106.70 (87.26, 131.68) ^***###$$$^ | 0.000 |
| eGFR4 | 173.03 (141.79, 209.01) | 160.13 (129.82, 197.83) ^***^ | 143.16 (116.59, 176.77) ^***###^ | 139.96 (114.97, 172.58) ^***###$$$^ | 0.000 |
| eGFR5 | 120.74 (108.12, 134.32) | 117.06 (104.07, 130.51) ^***^ | 111.71 (99.20, 124.58) ^***###^ | 110.70 (98.35, 123.61) ^***###$$$^ | 0.000 |
| eGFR6 | 142.76 (120.72, 170.74) | 135.08 (113.31, 162.61) ^***^ | 126.47 (106.58, 151.25) ^***###^ | 125.04 (104.89, 149.88) ^***###$$^ | 0.000 |
| eGFR7 | 149.30 (115.82, 198.67) | 142.52 (108.75, 189.96) ^***^ | 131.56 (100.32, 175.74) ^***###^ | 129.40 (99.05, 173.25) ^***###$$^ | 0.000 |
| eGFR8 | 111.35 (97.43, 129.29) | 109.89 (95.37, 126.08) ^***^ | 107.24 (93.20, 122.43) ^***###^ | 106.83 (92.82, 121.81) ^***###$^ | 0.000 |
| eGFR9 | 123.54 (110.77,134.65) | 120.09 (106.86,132.61) ^***^ | 115.09(101.37,128.10) ^***###^ | 114.03 (100.35,127.38) ^***###$$$^ | 0.000 |
| eGFR10 | 117.85(107.85,126.76) | 115.44(104.65,125.45) ^***^ | 111.58 (99.99,122.25) ^***###^ | 110.81 (99.08,121.72) ^***###$$$^ | 0.000 |

BMI, body mass index (calculated as weight/height^2^ ); Scr, serum creatinine; eGFR, estimated glomerular filtration rate. vs. underweight group, ^***^*p*＜0.001; vs. Normal weight group, ^###^*p*＜0.001; vs. Over weight group, ^$^*p*＜0.05, ^$$^*p*＜0.01, ^$$$^*p*＜0.001.

Table S13 Distribution across eGFR categories in populations with different weight levels

| Stage | Group | eGFR1 | eGFR2 | eGFR3 | eGFR4 | eGFR5 | eGFR6 | eGFR7 | eGFR8 | eGFR9 | eGFR10 |
| --- | --- | --- | --- | --- | --- | --- | --- | --- | --- | --- | --- |
| G1, n (%) | Under weight | 1683  (66.08) | 2306  (90.54) | 2311  (90.73) | 2515  (98.74) | 2427  (95.29) | 2440  (95.80) | 2337  (91.76) | 2148  (84.33) | 2442  (95.88) | 2437  (95.682) |
|  | Normal weight | 26354  (82.21) | 28188  (87.94) | 26802  (83.61) | 31135  (97.13) | 29622  (92.41) | 30228  (94.30) | 28286  (88.24) | 26420  (82.42) | 29691  (92.63) | 29583  (92.29) |
|  | Over weight | 26233  (89.74) | 23382  (79.99) | 21680  (74.17) | 27686  (94.71) | 25779  (88.19) | 26806  (91.70) | 24412  (83.51) | 23386  (80.00) | 25649  (87.74) | 25495  (87.22) |
|  | Obesity | 11190  (95.32) | 9222  (78.56) | 8430  (71.81) | 11041  (94.05) | 10194  (86.84) | 10708  (91.22) | 9688  (82.53) | 9307  (79.28) | 10179  (86.71) | 10110  (86.12) |
| G2, n (%) | Under weight | 778  (30.55) | 176  (6.91) | 224  (8.79) | 28  (1.10) | 115  (4.52) | 103  (4.04) | 193  (7.58) | 390  (15.31) | 99  (3.89) | 105  (4.12) |
|  | Normal weight | 5235  (16.33) | 3739  (11.66) | 4973  (15.51) | 893  (2.79) | 2361  (7.37) | 1799  (5.61) | 3505  (10.93) | 5479  (17.09) | 2290  (7.14) | 2397  (7.48) |
|  | Over weight | 2769  (9.47) | 5566  (19.04) | 7055  (24.13) | 1470  (5.03) | 3330  (11.39) | 2359  (8.07) | 4385  (15.00) | 5635  (19.28) | 3394  (11.61) | 3553  (12.15) |
|  | Obesity | 518(4.41) | 2389  (20.35) | 3103  (26.43) | 670  (5.71) | 1501  (12.79) | 1007  (8.58) | 1880  (16.01) | 2343  (19.96) | 1493  (12.72) | 1562  (13.31) |
| G3a, n (%) | Under weight | 70  (2.75) | 2  (0.08) | 8  (0.31) | 1  (0.04) | 2  (0.08) | 2  (0.08) | 14  (0.55) | 8  (0.31) | 3  (0.12) | 2  (0.08) |
|  | Normal weight | 396  (1.24) | 104  (0.32) | 250  (0.78) | 11  (0.03) | 58  (0.18) | 17(0.05) | 242(0.75) | 143  (0.45) | 52  (0.16) | 53  (0.17) |
|  | Over weight | 181  (0.62) | 225  (0.77) | 416  (1.42) | 40  (0.14) | 97  (0.33) | 40  (0.14) | 394  (1.35) | 186  (0.64) | 134  (0.46) | 131  (0.45) |
|  | Obesity | 24  (0.20) | 111  (0.95) | 184  (1.57) | 20  (0.17) | 38  (0.32) | 14  (0.12) | 155  (1.32) | 83  (0.71) | 55  (0.47) | 55  (0.47) |
| G3b, n (%) | Under weight | 12  (0.47) | 0  (0.00) | 2  (0.08) | 1(0.04) | 2  (0.08) | 1  (0.04) | 1  (0.04) | 0  (0.00) | 0  (0.00) | 0  (0.00) |
|  | Normal weight | 58  (0.18) | 14  (0.04) | 19  (0.06) | 8  (0.02) | 8  (0.02) | 4  (0.01) | 16  (0.05) | 7  (0.02) | 12  (0.04) | 12  (0.04) |
|  | Over weight | 34  (0.12) | 34  (0.12) | 57  (0.19) | 21  (0.07) | 14  (0.05) | 13  (0.04) | 31  (0.11) | 15  (0.05) | 33  (0.11) | 31  (0.11) |
|  | Obesity | 5  (0.04) | 12  (0.10) | 18  (0.15) | 6  (0.05) | 4  (0.03) | 8  (0.07) | 13  (0.11) | 4  (0.03) | 7  (0.06) | 7  (0.06) |
| G4, n (%) | Under weight | 2  (0.08) | 1  (0.04) | 0  (0.00) | 1  (0.04) | 1  (0.04) | 0  (0.00) | 2  (0.08) | 1  (0.04) | 1  (0.04) | 1  (0.04) |
|  | Normal weight | 8  (0.02) | 4  (0.01) | 5  (0.02) | 3  (0.01) | 1  (0.00) | 2  (0.01) | 4  (0.01) | 3  (0.01) | 5  (0.02) | 5  (0.02) |
|  | Over weight | 9  (0.03) | 18  (0.063) | 17  (0.06) | 9  (0.03) | 11  (0.00) | 8  (0.03) | 8  (0.03) | 9  (0.03) | 15  (0.05) | 15  (0.05) |
|  | Obesity | 1  (0.01) | 3  (0.03) | 2  (0.02) | 0  (0.00) | 2  (0.02) | 2  (0.02) | 2  (0.02) | 2  (0.02) | 3  (0.03) | 3  (0.03) |
| G5, n (%) | Under weight | 2  (0.08) | 2  (0.08) | 2  (0.08) | 1  (0.04) | 0  (0.00) | 1  (0.04) | 0  (0.00) | 0  (0.00) | 2  (0.08) | 2  (0.08) |
|  | Normal weight | 4  (0.01) | 6  (0.02) | 6  (0.02) | 5  (0.02) | 5  (0.02) | 5  (0.02) | 2  (0.01) | 3  (0.01) | 5  (0.02) | 5  (0.02) |
|  | Over weight | 6  (0.02) | 7  (0.027) | 7  (0.02) | 6  (0.02) | 1  (0.00) | 6  (0.02) | 2  (0.01) | 1  (0.00) | 7  (0.02) | 7  (0.02) |
|  | Obesity | 1  (0.01) | 2  (0.02) | 2  (0.02) | 2  (0.02) | 0  (0.00) | 0  (0.00) | 1  (0.01) | 0  (0.00) | 2  (0.02) | 2  (0.02) |

eGFR, estimated glomerular filtration rate.

Table S14 Concordance among equations in underweight group

| Equation | | eGFR1 | eGFR2 | eGFR3 | eGFR4 | eGFR5 | eGFR6 | eGFR7 | eGFR8 | eGFR9 | eGFR10 | |
| --- | --- | --- | --- | --- | --- | --- | --- | --- | --- | --- | --- | --- |
|  | eGFR1 | 1.000 |  |  |  |  |  |  |  |  |  |  |
|  | eGFR2 | 0.212(0.176-0.247) | 1.000 |  |  |  |  |  |  |  |  |  |
|  | eGFR3 | 0.228(0.191-0.264) | 0.409(0.339-0.479) | 1.000 |  |  |  |  |  |  |  |  |
|  | eGFR4 | 0.06(0.033-0.088) | 0.343(0.242-0.444) | 0.226(0.138-0.314) | 1.000 |  |  |  |  |  |  |  |
|  | eGFR5 | 0.173(0.144-0.202) | 0.336(0.259-0.413) | 0.33(0.263-0.397) | 0.256(0.148-0.363) | 1.000 |  |  |  |  |  |  |
|  | eGFR6 | 0.09(0.061-0.119) | 0.2(0.116-0.284) | 0.183(0.109-0.256) | 0.151(0.016-0.286) | 0.239(0.156-0.322) | 1.000 |  |  |  |  |  |
|  | eGFR7 | 0.298(0.265-0.331) | 0.313(0.244-0.382) | 0.341(0.276-0.405) | 0.203(0.114-0.293) | 0.593(0.533-0.652) | 0.147(0.075-0.218) | 1.000 |  |  |  |  |
|  | eGFR8 | 0.343(0.309-0.376) | 0.163(0.112-0.214) | 0.152(0.103-0.201) | 0.081(0.039-0.123) | 0.4(0.345-0.454) | 0.062(0.018-0.106) | 0.465(0.416-0.514) | 1.000 |  |  |  |
|  | eGFR9 | 0.159(0.125-0.193) | 0.661(0.592-0.731) | 0.367(0.289-0.445) | 0.514(0.399-0.628) | 0.471(0.388-0.554) | 0.209(0.108-0.311) | 0.421(0.35-0.493) | 0.216(0.163-0.268) | 1.000 |  |  |
|  | eGFR10 | 0.162(0.128-0.196) | 0.685(0.618-0.751) | 0.372(0.295-0.45) | 0.501(0.386-0.616) | 0.47(0.388-0.553) | 0.204(0.104-0.305) | 0.397(0.326-0.469) | 0.212(0.159-0.264) | 0.948(0.919-0.978) | 1.000 |  |

eGFR, estimated glomerular filtration rate.

Table S15 Concordance among equations in normal weight group

| Equation | eGFR1 | eGFR2 | eGFR3 | eGFR4 | eGFR5 | eGFR6 | eGFR7 | eGFR8 | eGFR9 | eGFR10 |
| --- | --- | --- | --- | --- | --- | --- | --- | --- | --- | --- |
| eGFR1 | 1.000 |  |  |  |  |  |  |  |  |  |
| eGFR2 | 0.312(0.298-0.325) | 1.000 |  |  |  |  |  |  |  |  |
| eGFR3 | 0.339(0.326-0.352) | 0.434(0.421-0.448) | 1.000 |  |  |  |  |  |  |  |
| eGFR4 | 0.171(0.158-0.184) | 0.361(0.344-0.379) | 0.23(0.216-0.245) | 1.000 |  |  |  |  |  |  |
| eGFR5 | 0.453(0.441-0.466) | 0.336(0.319-0.352) | 0.318(0.304-0.332) | 0.255(0.234-0.276) | 1.000 |  |  |  |  |  |
| eGFR6 | 0.172(0.16-0.184) | 0.176(0.161-0.191) | 0.171(0.158-0.184) | 0.195(0.173-0.216) | 0.309(0.29-0.328) | 1.000 |  |  |  |  |
| eGFR7 | 0.653(0.642-0.664) | 0.323(0.308-0.338) | 0.335(0.321-0.348) | 0.211(0.194-0.227) | 0.592(0.578-0.606) | 0.175(0.16-0.19) | 1.000 |  |  |  |
| eGFR8 | 0.516(0.504-0.528) | 0.198(0.185-0.212) | 0.208(0.195-0.221) | 0.105(0.093-0.116) | 0.509(0.496-0.523) | 0.115(0.103-0.127) | 0.564(0.552-0.577) | 1.000 |  |  |
| eGFR9 | 0.347(0.334-0.361) | 0.652(0.638-0.667) | 0.349(0.334-0.363) | 0.548(0.527-0.569) | 0.446(0.428-0.464) | 0.193(0.175-0.211) | 0.413(0.397-0.429) | 0.255(0.241-0.269) | 1.000 |  |
| eGFR10 | 0.347(0.334-0.361) | 0.67(0.656-0.683) | 0.35(0.335-0.364) | 0.53(0.509-0.55) | 0.445(0.427-0.463) | 0.198(0.18-0.216) | 0.403(0.387-0.419) | 0.251(0.237-0.264) | 0.251(0.237-0.264) | 1.000 |

eGFR, estimated glomerular filtration rate.

Table S16 Concordance among equations in overweight group

| Equation | eGFR1 | eGFR2 | eGFR3 | eGFR4 | eGFR5 | eGFR6 | eGFR7 | eGFR8 | eGFR9 | eGFR10 | |
| --- | --- | --- | --- | --- | --- | --- | --- | --- | --- | --- | --- |
| eGFR1 | 1.000 |  |  |  |  |  |  |  |  |  |  |
| eGFR2 | 0.298(0.283-0.312) | 1.000 |  |  |  |  |  |  |  |  |  |
| eGFR3 | 0.283(0.271-0.296) | 0.405(0.393-0.418) | 1.000 |  |  |  |  |  |  |  |  |
| eGFR4 | 0.327(0.307-0.347) | 0.376(0.362-0.39) | 0.243(0.231-0.255) | 1.000 |  |  |  |  |  |  |  |
| eGFR5 | 0.615(0.601-0.629) | 0.323(0.309-0.337) | 0.291(0.278-0.303) | 0.276(0.258-0.293) | 1.000 |  |  |  |  |  |  |
| eGFR6 | 0.259(0.241-0.277) | 0.208(0.194-0.222) | 0.176(0.165-0.188) | 0.245(0.223-0.266) | 0.366(0.349-0.382) | 1.000 |  |  |  |  |  |
| eGFR7 | 0.64(0.628-0.652) | 0.326(0.312-0.339) | 0.332(0.32-0.344) | 0.248(0.233-0.263) | 0.603(0.591-0.615) | 0.208(0.194-0.222) | 1.000 |  |  |  |  |
| eGFR8 | 0.45(0.436-0.463) | 0.225(0.212-0.238) | 0.223(0.211-0.236) | 0.154(0.141-0.166) | 0.621(0.609-0.633) | 0.183(0.17-0.196) | 0.621(0.61-0.632) | 1.000 |  |  |  |
| eGFR9 | 0.453(0.438-0.469) | 0.666(0.655-0.678) | 0.339(0.327-0.352) | 0.578(0.562-0.594) | 0.447(0.432-0.462) | 0.237(0.22-0.254) | 0.432(0.418-0.446) | 0.31(0.296-0.323) | 1.000 |  |  |
| eGFR10 | 0.439(0.424-0.455) | 0.687(0.675-0.698) | 0.342(0.329-0.355) | 0.56(0.544-0.577) | 0.442(0.427-0.457) | 0.244(0.227-0.261) | 0.415(0.401-0.429) | 0.301(0.288-0.315) | 0.965(0.961-0.97) | 1.000 |  |

eGFR, estimated glomerular filtration rate.

Table S17 Concordance among equations in obesity group

| Equation | eGFR1 | eGFR2 | eGFR3 | eGFR4 | eGFR5 | eGFR6 | eGFR7 | eGFR8 | eGFR9 | eGFR10 |
| --- | --- | --- | --- | --- | --- | --- | --- | --- | --- | --- |
| eGFR1 | 1.000 |  |  |  |  |  |  |  |  |  |
| eGFR2 | 0.177(0.157-0.197) | 1.000 |  |  |  |  |  |  |  |  |
| eGFR3 | 0.16(0.144-0.177) | 0.406(0.387-0.424) | 1.000 |  |  |  |  |  |  |  |
| eGFR4 | 0.301(0.264-0.339) | 0.379(0.358-0.399) | 0.237(0.22-0.254) | 1.000 |  |  |  |  |  |  |
| eGFR5 | 0.428(0.402-0.453) | 0.302(0.281-0.322) | 0.291(0.273-0.309) | 0.256(0.23-0.281) | 1.000 |  |  |  |  |  |
| eGFR6 | 0.191(0.16-0.221) | 0.17(0.149-0.19) | 0.16(0.143-0.178) | 0.223(0.193-0.253) | 0.345(0.32-0.37) | 1.000 |  |  |  |  |
| eGFR7 | 0.361(0.339-0.383) | 0.312(0.292-0.332) | 0.326(0.308-0.344) | 0.238(0.215-0.261) | 0.619(0.601-0.637) | 0.189(0.168-0.21) | 1.000 |  |  |  |
| eGFR8 | 0.257(0.237-0.276) | 0.22(0.2-0.24) | 0.23(0.212-0.249) | 0.144(0.125-0.163) | 0.652(0.634-0.67) | 0.178(0.157-0.198) | 0.639(0.622-0.656) | 1.000 |  |  |
| eGFR9 | 0.338(0.311-0.365) | 0.658(0.64-0.675) | 0.331(0.312-0.35) | 0.587(0.563-0.611) | 0.412(0.389-0.435) | 0.186(0.161-0.211) | 0.422(0.401-0.443) | 0.3(0.279-0.321) | 1.000 |  |
| eGFR10 | 0.323(0.297-0.349) | 0.68(0.663-0.697) | 0.336(0.317-0.355) | 0.567(0.544-0.591) | 0.41(0.387-0.432) | 0.19(0.165-0.214) | 0.405(0.384-0.427) | 0.292(0.271-0.313) | 0.964(0.957-0.971) | 1.000 |

eGFR, estimated glomerular filtration rate.

Table S18 The baseline data of participants with rGFR in sexual groups

| Characteristic | Male (n=419) | Female (n=332) | *p*-Value |
| --- | --- | --- | --- |
| Age (years) | 58.00 (50.00,69.00) | 57.00(50.00, 67.00) | 0.370 |
| BMI (kg/m^2^) | 23.91 ± 3.39 | 23.73 (21.48, 26.21) | 0.701 |
| Scr (mg/dL) | 0.95 (0.79, 1.18) | 0.68 (0.57, 0.83) | 0.000 |
| rGFR | 66.84 ± 24.91 | 68.24 ± 23.66 | 0.437 |
| eGFR |  |  |  |
| eGFR1 | 80.52(58.57, 106.06) | 84.18(60.85, 107.95) | 0.201 |
| eGFR2 | 91.68 (70.16, 110.59) | 91.09 (68.09, 113.57) | 0.777 |
| eGFR3 | 86.11 (66.01, 104.73) | 86.16 (63.79, 105.18) | 0.609 |
| eGFR4 | 112.85 (86.51, 137.25) | 112.91 (83.6, 137.84) | 0.609 |
| eGFR5 | 89.98 (67.18, 101.75) | 92.44 (70.93, 104.08) | 0.133 |
| eGFR6 | 104.65 (83.31, 125.34) | 106.83 (86.3, 126.83) | 0.410 |
| eGFR7 | 84.55 (66.65, 106.17) | 87.98 (66.68, 110.67) | 0.153 |
| eGFR8 | 105.05 (79.95, 112.35) | 105.9 (84.11, 113.57) | 0.300 |
| eGFR9 | 99.98 (79.56,109.40) | 92.87 (68.28,107.03) | 0.002 |
| eGFR10 | 99.02 (79.68,107.14) | 92.84 (69.07,105.08) | 0.001 |

BMI, body mass index (calculated as weight/height^2^ ); Scr, serum creatinine; rGFR, reference glomerular filtration rate; eGFR, estimated glomerular filtration rate.

Table S19 The baseline data of participants with rGFR in different age groups

| Characteristic | Adults aged 18-39 years  (n=68) | Adults aged 40-60 years (n=364) | Adults aged ＞60 years (n=318) | *p*-Value |
| --- | --- | --- | --- | --- |
| Sex (male/female) | 43/25 | 194/170 | 181/137^***^ | 0.000 |
| BMI (kg/m^2^) | 23.35 (20.73, 25.17) | 24.39 (22.04, 26.82) | 23.44 (20.6, 25.61) | 0.791 |
| Scr (mg/dL) | 0.80 (0.63, 0.96) | 0.79 (0.66, 1.01) | 0.86 (0.70, 1.16) ^#^ | 0.002 |
| rGFR | 87.08 ± 26.32 | 71.39 ± 23.61^***^ | 58.77 ± 21.00^***###^ | 0.000 |
| eGFR |  |  |  |  |
| eGFR1 | 122.77 (108.09, 138.30) | 93.52 (72.87, 113.40) ^***^ | 62.37 (46.38, 81.38) ^***###^ | 0.000 |
| eGFR2 | 90.46 (69.94, 108.68) | 91.66 (70.85, 111.07) | 91.5 (67.25, 113.15) | 0.882 |
| eGFR3 | 92.51 (77.99, 103.53) | 83.48 (61.32, 105.05) | 88.06 (66.11, 105.40) | 0.167 |
| eGFR4 | 121.23 (102.21, 135.68) | 109.4 (80.36, 137.67) | 115.4 (86.63, 138.13) | 0.167 |
| eGFR5 | 92.54 (70.49, 103.05) | 91.71 (70.09, 102.28) | 89.33 (66.60, 103.13) | 0.774 |
| eGFR6 | 105.32 (86.81, 130.28) | 107.44 (84.63, 127.08) | 103.91 (82.98, 123.75) | 0.489 |
| eGFR7 | 89.37 (68.95, 115.45) | 85.85 (67.93, 106.97) | 86.41 (63.95, 114.26) | 0.736 |
| eGFR8 | 107.37 (86.99, 113.39) | 107.61 (83.11, 113.82) | 102.72 (80.08, 112.34) | 0.204 |
| eGFR9 | 124.44(111.37,131.88) | 104.47(80.71,111.47) ^***^ | 86.55 (58.73,97.33) ^***###^ | 0.000 |
| eGFR10 | 120.50(108.80,126.10) | 103.45 (79.85,108.88) ^***^ | 87.75 (59.39,96.71) ^***###^ | 0.000 |

BMI, body mass index (calculated as weight/height^2^ ); Scr, serum creatinine；rGFR, reference glomerular filtration rate; eGFR, estimated glomerular filtration rate. vs. adults aged 18-39 years group, ^***^*p*＜0.001; vs. adults aged 40-60 years, ^#^*p*＜0.05, ^###^*p*＜0.001.

Table S20 The baseline data of participants with rGFR in different weight groups

| Characteristic | Underweight (n=39) | Normal weight (n=344) | Overweight (n=283) | Obesity (n=84) | *p*-Value |
| --- | --- | --- | --- | --- | --- |
| Sex (male/female) | 23/16 | 183/161 | 166/117 | 46/38 | 0.557 |
| Age (years) | 54.00 (68.00, 75.00) | 51.00 (58.00, 69.00) ^**^ | 49.00 (56.00, 67.00) ^***^ | 50.25 (55.5, 65) ^***^ | 0.001 |
| Scr (mg/dL) | 0.61 (0.77, 1.04) | 0.66 (0.79, 1.03) | 0.70 (0.86, 1.07) | 0.70 (0.88, 1.10) | 0.050 |
| rGFR | 58.44 ± 26.32 | 76.87 ± 31.93 | 89.39 ± 32.62 | 100.07 ± 38.6 | 0.160 |
| eGFR |  |  |  |  |  |
| eGFR1 | 51.48 (40.74, 70.63) | 74.72 (57.33, 96.98) ^***^ | 90.23 (66.42, 110.13) ^***###^ | 106.74 (77.63, 128.67) ^***###$$$^ | 0.000 |
| eGFR2 | 99.5 (65.59, 121.91) | 92.62 (70.03, 113.84) | 90.76 (68.47, 109.67) | 89.4 (68.31, 108.99) | 0.329 |
| eGFR3 | 85.12 (65.78, 112.36) | 86.16 (67.47, 107.26) | 87.33 (63.75, 103.49) | 83.25 (58.78, 98.21) | 0.423 |
| eGFR4 | 111.55 (86.20, 147.24) | 112.91 (88.42, 140.56) | 114.45 (83.54, 135.63) | 109.09 (77.03, 128.70) | 0.423 |
| eGFR5 | 82.9 (71.04, 99.66) | 91.12 (71.42, 103.56) | 91.94 (64.71, 103.03) | 90.3 (70.68, 100.85) | 0.832 |
| eGFR6 | 101.75 (86.14, 120.75) | 106.73 (86.54, 125.53) | 106.07 (81.97, 128.93) | 102.86 (85.36, 120.23) | 0.882 |
| eGFR7 | 76.67 (67, 101.37) | 86.56 (68.29, 109.72) | 87.18 (63.13, 109.34) | 85.14 (68.28, 103.65) | 0.847 |
| eGFR8 | 106.63 (88.85, 112.92) | 104.55 (83.39, 112.58) | 105.9 (79.55, 113.55) | 106.42 (82.01, 113.08) | 0.988 |
| eGFR9 | 91.70 (74.28,105.79) | 97.33 (70.35,110.37) | 95.36 (73.31,106.60) | 94.88(74.99,107.69) | 0.444 |
| eGFR10 | 91.69(75.31,103.26) | 96.75 (70.66,107.50) | 94.29(73.710,104.65) | 94.01 (74.82,105.81) | 0.445 |

Scr, serum creatinine: rGFR, reference glomerular filtration rate; eGFR, estimated glomerular filtration rate. vs. underweight group, ^**^*p*＜0.01, ^***^*p*＜0.001; vs. normal weight group, ^###^*p*＜0.001; vs. Over weight group, ^$$$^*p*＜0.001.

| Equation | ICC (95%CI) | γ | 𝛋 (95%CI) | SE | P10 (%) | P30 (%) | Absolute bias |
| --- | --- | --- | --- | --- | --- | --- | --- |
| eGFR1 | 0.518(0.417-0.603) | 0.625 | 0.402(0.332-0.472) | 31.06 | 18.07 | 50.90 | 18.65(8.07, 36.69) |
| eGFR2 | 0.013(-0.069-0.099) | 0.009 | 0.015(-0.043-0.072) | 45.52 | 12.35 | 37.651 | 31.53(14.81, 52.79) |
| eGFR3 | -0.011(-0.103-0.084) | -0.013 | 0.016(-0.054-0.086) | 43.89 | 12.65 | 40.06 | 31.17(14.22, 51.43) |
| eGFR4 | -0.007(-0.067-0.060) | -0.013 | -0.008(-0.067-0.050) | 65.00 | 8.43 | 21.08 | 49.88(25.61, 78.53) |
| eGFR5 | 0.082(-0.011-0.177) | 0.082 | 0.069(0.003-0.134) | 38.16 | 12.35 | 39.16 | 29.81(13.37, 46.82) |
| eGFR6 | 0.058(-0.027-0.147) | 0.106 | 0.061(0.007-0.116) | 55.36 | 9.64 | 28.31 | 42.14(19.46, 65.26) |
| eGFR7 | 0.039(-0.048-0.128) | 0.059 | 0.059(-0.007-0.126) | 56.66 | 9.94 | 35.24 | 31.39(15.77, 52.70) |
| eGFR8 | 0.077(-0.019-0.175) | 0.125 | 0.061(0.007-0.115) | 42.56 | 11.45 | 30.72 | 35.86(16.88, 52.52) |
| eGFR9 | 0.445(0.224-0.604) | 0.645 | 0.293(0.230-0.357) | 32.43 | 12.35 | 38.25 | 26.14(12.35, 40.22) |
| eGFR10 | 0.446(0.236-0.599) | 0.641 | 0.285(0.222-0.348) | 31.09 | 14.16 | 41.27 | 24.74(11.40, 38.41) |

Table S21 Concordance analysis of eGFR equations and rGFR in female group

eGFR, estimated glomerular filtration rate; ICC, interclass correlation coefficient; SE, Std. Error.

Table S22 Concordance analysis of eGFR equations and rGFR in male group

| Equation | ICC (95%CI) | γ | 𝛋 (95%CI) | SE | P10 (%) | P30 (%) | Absolute bias |
| --- | --- | --- | --- | --- | --- | --- | --- |
| eGFR1 | 0.542(0.465-0.610) | 0.643 | 0.442(0.380-0.504) | 22.65 | 15.27 | 52.98 | 18.71(9.10, 34.34) |
| eGFR2 | -0.11(-0.223-0.004) | -0.143 | -0.053(-0.102--0.003) | 40.49 | 11.93 | 31.74 | 36.07(17.77, 61.37) |
| eGFR3 | -0.025(-0.104-0.056) | -0.025 | 0.002(-0.050-0.054) | 35.05 | 15.27 | 35.08 | 32.11(14.32, 49.46) |
| eGFR4 | -0.015(-0.069-0.043) | -0.025 | 0.004(-0.035-0.044) | 51.78 | 6.44 | 23.63 | 50.23(25.15, 77.58) |
| eGFR5 | -0.051(-0.134-0.035) | -0.051 | -0.017(-0.072-0.037) | 31.07 | 11.69 | 34.13 | 31.07(16.85, 50.32) |
| eGFR6 | -0.033(-0.099-0.039) | -0.046 | -0.02(-0.062-0.023) | 42.08 | 9.31 | 26.49 | 44.13(21.92, 67.05) |
| eGFR7 | -0.049(-0.131-0.036) | -0.047 | -0.011(-0.067-0.046) | 41.25 | 10.98 | 34.37 | 31.25(16.39, 53.97) |
| eGFR8 | -0.034(-0.103-0.040) | -0.059 | -0.015(-0.059-0.028) | 34.67 | 10.26 | 30.07 | 38.22(20.25, 56.15) |
| eGFR9 | 0.488(0.370-0.585) | 0.634 | 0.352(0.289-0.414) | 23.04 | 15.99 | 46.78 | 22.66(10.07, 36.19) |
| eGFR10 | 0.495(0.383-0.587) | 0.632 | 0.355(0.292-0.418) | 22.10 | 16.71 | 48.45 | 21.83(9.71, 34.44) |

eGFR, estimated glomerular filtration rate; ICC, interclass correlation coefficient; SE, Std. Error.

Table S23 Concordance analysis of eGFR equations and rGFR in 18-39 years group

| Equation | ICC (95%CI) | γ | 𝛋 (95%CI) | SE | P10 (%) | P30 (%) | Absolute bias |
| --- | --- | --- | --- | --- | --- | --- | --- |
| eGFR1 | 0.347(0.053-0.580) | 0.536 | 0.202(-0.008-0.412) | 43.17 | 7.35 | 26.47 | 39.26 ± 18.07 |
| eGFR2 | -0.017(-0.258-0.224) | 0.023 | -0.105(-0.218-0.009) | 43.41 | 7.35 | 47.06 | 26.23(17.77, 47.75) |
| eGFR3 | -0.071(-0.307-0.172) | -0.125 | -0.079(-0.202-0.045) | 44.54 | 20.59 | 44.12 | 33.70(14.05, 49.23) |
| eGFR4 | -0.047(-0.217-0.146) | -0.125 | -0.035(-0.147-0.077) | 61.88 | 13.24 | 38.24 | 40.82(16.21, 79.51) |
| eGFR5 | 0.007(-0.235-0.246) | -0.005 | 0.017(-0.165-0.199) | 36.00 | 19.12 | 52.94 | 25.17(10.96, 38.36) |
| eGFR6 | 0.015(-0.175-0.219) | -0.025 | -0.01(-0.162-0.142) | 45.47 | 17.65 | 52.94 | 24.17(12.17, 57.11) |
| eGFR7 | -0.026(-0.265-0.215) | -0.009 | 0.043(-0.138-0.224) | 41.58 | 19.12 | 45.59 | 29.28(12.57, 45.30) |
| eGFR8 | 0.036(-0.187-0.261) | 0.004 | 0.022(-0.166-0.211) | 36.00 | 17.65 | 58.82 | 21.61(13.11, 45.78) |
| eGFR9 | 0.258(0.009-0.483) | 0.354 | 0.156(-0.042-0.354) | 40.68 | 11.76 | 32.35 | 35.77 ± 19.50 |
| eGFR10 | 0.272(0.038-0.482) | 0.356 | 0.156(-0.042-0.354) | 37.15 | 16.18 | 39.71 | 32.14 ± 18.76 |

eGFR, estimated glomerular filtration rate; ICC, interclass correlation coefficient; SE, Std. Error.

Table S24 Concordance analysis of eGFR equations and rGFR in 40-60 years group

| Equation | ICC (95%CI) | γ | 𝛋 (95%CI) | SE | P10 (%) | P30 (%) | Absolute bias |
| --- | --- | --- | --- | --- | --- | --- | --- |
| eGFR1 | 0.347(0.053-0.580) | 0.598 | 0.202(-0.008~ 0.412) | 33.23 | 14.84 | 47.53 | 22.99(10.59, 38.97) |
| eGFR2 | -0.017(-0.258-0.224) | -0.036 | -0.105(-0.218~ 0.009) | 46.86 | 14.29 | 38.74 | 31.27(14.49, 51.08) |
| eGFR3 | -0.071(-0.307-0.172) | -0.039 | -0.079(-0.202~ 0.045) | 42.48 | 14.29 | 42.86 | 28.96(13.32, 47.73) |
| eGFR4 | -0.047(-0.217-0.146) | -0.039 | -0.035(-0.147~ 0.077) | 62.39 | 8.24 | 28.30 | 42.83(21.61, 70.50) |
| eGFR5 | 0.007(-0.235-0.246) | -0.088 | 0.017(-0.165~ 0.199) | 39.56 | 12.36 | 40.11 | 28.93(16.15, 46.52) |
| eGFR6 | 0.015(-0.175-0.219) | -0.068 | -0.01(-0.162~ 0.142) | 55.19 | 9.89 | 29.67 | 42.00(18.59, 65.77) |
| eGFR7 | -0.026(-0.265-0.215) | -0.092 | 0.043(-0.138~ 0.224) | 52.55 | 9.89 | 37.91 | 29.99(17.41, 52.30) |
| eGFR8 | 0.036(-0.187-0.261) | -0.089 | 0.022(-0.166~ 0.211) | 44.26 | 11.54 | 33.24 | 36.22(16.88, 52.61) |
| eGFR9 | 0.258(0.009-0.483) | 0.617 | 0.156(-0.042~ 0.354) | 31.88 | 14.84 | 45.60 | 25.60(12.04, 40.13) |
| eGFR10 | 0.272(0.038-0.482) | 0.611 | 0.156(-0.042~ 0.354) | 30.31 | 16.49 | 48.35 | 23.44(11.18, 38.01) |

eGFR, estimated glomerular filtration rate; ICC, interclass correlation coefficient; SE, Std. Error.

Table S25 Concordance analysis of eGFR equations and rGFR in ＞60 years group

| Equation | ICC (95%CI) | γ | 𝛋 (95% CI) | SE | P10 (%) | P30 (%) | Absolute bias |
| --- | --- | --- | --- | --- | --- | --- | --- |
| eGFR1 | 0.502(0.416~ 0.579) | 0.554 | 0.412(0.340~ 0.483) | 23.55 | 20.44 | 62.89 | 12.08(6.70, 21.89) |
| eGFR2 | -0.075(-0.184~0.039) | -0.132 | -0.042(-0.088~0.004) | 55.80 | 10.69 | 26.73 | 39.76(19.67, 65.19) |
| eGFR3 | 0.002(-0.072~0.082) | 0.02 | 0.027(-0.031~0.085) | 49.17 | 12.58 | 29.56 | 35.46(16.86, 53.31) |
| eGFR4 | 0.001(-0.048~0.057) | 0.02 | -0.001(-0.046~0.045) | 73.90 | 5.03 | 12.58 | 57.07(32.00, 80.51) |
| eGFR5 | 0.065(-0.024~0.157) | 0.121 | 0.028(-0.026~0.082) | 41.58 | 10.06 | 28.62 | 32.61(16.41, 53.20) |
| eGFR6 | 0.039(-0.032~0.116) | 0.103 | 0.022(-0.019~0.063) | 58.71 | 7.23 | 19.18 | 46.46(25.59, 69.99) |
| eGFR7 | 0.049(-0.035~0.136) | 0.114 | 0.042(-0.013~0.097) | 61.93 | 9.43 | 28.93 | 34.19(16.25, 57.15) |
| eGFR8 | 0.048(-0.032~0.132) | 0.124 | 0.027(-0.015~0.068) | 46.98 | 8.49 | 21.07 | 41.23(23.28, 57.19) |
| eGFR9 | 0.417(0.268~ 0.538) | 0.549 | 0.306(0.240~ 0.372) | 28.86 | 14.47 | 42.45 | 21.59(9.59, 33.13) |
| eGFR10 | 0.413(0.263~ 0.536) | 0.547 | 0.303(0.237~ 0.369) | 28.55 | 14.47 | 43.08 | 21.22(9.71, 32.72) |

eGFR, estimated glomerular filtration rate; ICC, interclass correlation coefficient; SE, Std. Error.

Table S26 Concordance analysis of eGFR equations and rGFR in underweight group

| Equation | ICC (95%CI) | γ | 𝛋 (95% CI) | SE | P10 (%) | P30 (%) | Absolute bias |
| --- | --- | --- | --- | --- | --- | --- | --- |
| eGFR1 | 0.690(0.481-0.824) | 0.679 | 0.451(0.261~0.641) | 18.91 | 25.64 | 61.54 | 15.17 ± 11.44 |
| eGFR2 | -0.183(-0.469-0.136) | -0.209 | -0.072(-0.212~ 0.068) | 59.83 | 7.69 | 28.21 | 38.23(22.57, 74.34) |
| eGFR3 | -0.097(-0.397-0.221) | -0.072 | 0.004(-0.122~0.130) | 48.09 | 10.26 | 20.51 | 40.07 ± 26.94 |
| eGFR4 | -0.085(-0.387-0.233) | -0.072 | 0.000(-0.105~0.105) | 72.20 | 7.69 | 20.51 | 60.87 ± 39.33 |
| eGFR5 | -0.187(-0.472-0.132) | -0.194 | -0.111(-0.225~0.003) | 41.41 | 15.38 | 38.46 | 34.43 ± 23.30 |
| eGFR6 | -0.156(-0.446-0.164) | -0.175 | -0.061(-0.142~0.019) | 55.43 | 10.26 | 23.08 | 46.85 ± 30.02 |
| eGFR7 | -0.129(-0.424-0.191) | -0.196 | -0.122(-0.249~0.004) | 52.34 | 7.69 | 30.77 | 30.76(16.87, 52.69) |
| eGFR8 | -0.136(-0.430-0.184) | -0.116 | -0.046(-0.137~0.045) | 49.23 | 2.56 | 25.64 | 42.97 ± 24.34 |
| eGFR9 | 0.61(0.368-0.775) | 0.667 | 0.242(0.060~0.423) | 37.21 | 7.69 | 25.64 | 31.14 ± 20.63 |
| eGFR10 | 0.639(0.409-0.793) | 0.678 | 0.242(0.060~0.423) | 34.57 | 7.69 | 30.77 | 29.47 ± 18.30 |

eGFR, estimated glomerular filtration rate; ICC, interclass correlation coefficient; SE, Std. Error.

Table S27 Concordance analysis of eGFR equations and rGFR in normal weight group

| Equation | ICC (95%CI) | γ | 𝛋 (95%CI) | SE | P10 (%) | P30 (%) | Absolute bias |
| --- | --- | --- | --- | --- | --- | --- | --- |
| eGFR1 | 0.670(0.607-0.724) | 0.691 | 0.509(0.443~ 0.575) | 24.77 | 20.06 | 62.79 | 13.40(7.14, 27.32) |
| eGFR2 | -0.020(-0.125-0.086) | -0.009 | 0.028(-0.031~0.086) | 49.29 | 13.66 | 34.30 | 32.93(15.54, 56.83) |
| eGFR3 | -0.035(-0.140-0.071) | -0.021 | 0.011(-0.056~0.078) | 48.80 | 14.53 | 38.37 | 31.78(13.07, 52.09) |
| eGFR4 | -0.031(-0.136-0.075) | -0.021 | 0.002(-0.053~0.057) | 71.97 | 8.14 | 19.77 | 52.74(25.82, 79.93) |
| eGFR5 | 0.001(-0.104-0.107) | -0.015 | 0.025(-0.041~0.091) | 40.02 | 11.63 | 36.34 | 31.34(14.94, 48.56) |
| eGFR6 | 0.001(-0.105-0.106) | -0.003 | 0.018(-0.033~0.069) | 55.15 | 8.43 | 27.33 | 42.75(20.36, 67.97) |
| eGFR7 | -0.039(-0.144-0.067) | -0.030 | 0.011(-0.057~0.078) | 56.45 | 11.05 | 34.01 | 31.15(16.41, 55.38) |
| eGFR8 | 0.025(-0.081-0.130) | -0.002 | 0.017(-0.035~0.069) | 44.27 | 10.47 | 30.81 | 36.71(18.29, 54.02) |
| eGFR9 | 0.666(0.602-0.721) | 0.670 | 0.349(0.284~0.415) | 31.60 | 14.24 | 40.70 | 25.48(12.16, 38.56) |
| eGFR10 | 0.663(0.599-0.718) | 0.664 | 0.352(0.286~0.418) | 30.13 | 14.83 | 44.77 | 23.79(11.43, 35.94) |

eGFR, estimated glomerular filtration rate; ICC, interclass correlation coefficient; SE, Std. Error.

Table S28 Concordance analysis of eGFR equations and rGFR in overweight group

| Equation | ICC (95%CI) | γ | 𝛋 (95% CI) | SE | P10 (%) | P30 (%) | Absolute bias |
| --- | --- | --- | --- | --- | --- | --- | --- |
| eGFR1 | 0.597(0.517-0.667) | 0.617 | 0.326(0.249~0.403) | 33.34 | 13.07 | 44.17 | 25.18(11.26, 38.76) |
| eGFR2 | -0.111(-0.225-0.005) | -0.129 | -0.059(-0.117~-0.001) | 51.05 | 11.66 | 35.69 | 35.38(16.15, 54.60) |
| eGFR3 | 0.001(-0.115-0.117) | -0.009 | 0.015(-0.050~0.080) | 42.33 | 13.78 | 38.52 | 31.79(14.28, 47.66) |
| eGFR4 | 0.001(-0.115-0.117) | -0.009 | -0.002(-0.052~0.048) | 63.29 | 6.71 | 24.38 | 46.05(25.40, 73.19) |
| eGFR5 | 0.084(-0.033-0.198) | 0.101 | 0.072(0.004~0.140) | 39.78 | 12.37 | 37.46 | 29.71(14.46, 48.33) |
| eGFR6 | 0.081(-0.035-0.196) | 0.106 | 0.046(-0.011~0.103) | 57.40 | 10.95 | 27.56 | 43.47(20.08, 66.31) |
| eGFR7 | 0.048(-0.069-0.163) | 0.099 | 0.073(0.003~0.142) | 58.18 | 10.60 | 37.10 | 30.60(15.27, 53.37) |
| eGFR8 | 0.082(-0.035-0.197) | 0.091 | 0.05(-0.007~0.108) | 44.14 | 12.37 | 30.74 | 37.13(16.24, 53.02) |
| eGFR9 | 0.581(0.499-0.654) | 0.601 | 0.284(0.207~0.361) | 31.12 | 13.78 | 46.64 | 23.05(11.37, 38.56) |
| eGFR10 | 0.574(0.491-0.647) | 0.599 | 0.277(0.199~0.354) | 30.15 | 14.84 | 47.00 | 21.75(10.37, 36.84) |

eGFR, estimated glomerular filtration rate; ICC, interclass correlation coefficient; SE, Std. Error.

Table S29 Concordance analysis of eGFR equations and rGFR in obesity group

| Equation | ICC (95%CI) | γ | 𝛋 (95% CI) | SE | P10 (%) | P30 (%) | Absolute bias |
| --- | --- | --- | --- | --- | --- | --- | --- |
| eGFR1 | 0.582(0.421-0.708) | 0.629 | 0.393(0.266-0.520) | 43.85 | 9.52 | 30.95 | 33.00(15.26, 53.22) |
| eGFR2 | -0.143(-0.346-0.073) | -0.108 | -0.091(-0.198-0.017) | 48.99 | 9.52 | 33.33 | 30.08(16.62, 56.41) |
| eGFR3 | 0.000(-0.221-0.205) | -0.014 | -0.026(-0.157-0.104) | 41.37 | 15.48 | 36.90 | 31.30(14.40, 51.50) |
| eGFR4 | -0.008(-0.221-0.205) | -0.014 | -0.023(-0.123-0.077) | 59.24 | 5.95 | 28.57 | 49.56(22.45, 73.88) |
| eGFR5 | -0.187(-0.385-0.028) | -0.135 | -0.123(-0.226--0.020) | 40.89 | 10.71 | 32.14 | 31.43(19.32, 49.65) |
| eGFR6 | -0.178(-0.377-0.037) | -0.121 | -0.058(-0.150-0.034) | 54.16 | 8.33 | 28.57 | 43.76(23.07, 62.33) |
| eGFR7 | -0.11(-0.316-0.106) | -0.14 | -0.063(-0.167-0.040) | 46.41 | 9.52 | 32.14 | 33.69(13.96, 53.95) |
| eGFR8 | -0.146(-0.349-0.069) | -0.077 | -0.058(-0.150-0.034) | 46.55 | 10.71 | 29.76 | 40.29 ± 23.45 |
| eGFR9 | 0.612(0.458-0.730) | 0.614 | 0.422(0.299-0.545) | 29.76 | 20.24 | 48.81 | 19.14(7.95, 37.56) |
| eGFR10 | 0.609(0.455-0.728) | 0.616 | 0.422(0.299-0.545) | 28.90 | 2.50 | 48.81 | 18.57(7.29, 37.30) |

eGFR, estimated glomerular filtration rate; ICC, interclass correlation coefficient; SE, Std. Error.

Table S30 ROC curve analysis of eGFR equations based on rGFR＜90 ml/min·1.73m^2^ in 750 participants

| Equation | AUC | 95%CI | Std. Error | p-Value | YI | Sen | Spe |
| --- | --- | --- | --- | --- | --- | --- | --- |
| eGFR1 | 0.181 | 0.143-0.220 | 0.020 | 0.000 | 0.000 | 0.000 | 1.000 |
| eGFR2 | 0.517 | 0.463-0.571 | 0.028 | 0.525 | 0.064 | 0.882 | 0.181 |
| eGFR3 | 0.504 | 0.451-0.557 | 0.027 | 0.884 | 0.068 | 0.322 | 0.746 |
| eGFR4 | 0.504 | 0.451-0.557 | 0.027 | 0.884 | 0.068 | 0.322 | 0.746 |
| eGFR5 | 0.495 | 0.443-0.547 | 0.027 | 0.849 | 0.047 | 0.286 | 0.761 |
| eGFR6 | 0.493 | 0.441-0.545 | 0.027 | 0.797 | 0.044 | 0.377 | 0.667 |
| eGFR7 | 0.500 | 0.448-0.552 | 0.027 | 0.997 | 0.036 | 0.087 | 0.949 |
| eGFR8 | 0.474 | 0.422-0.526 | 0.027 | 0.333 | 0.045 | 0.082 | 0.964 |
| eGFR9 | 0.184 | 0.145-0.223 | 0.020 | 0.000 | 0.002 | 0.002 | 1.000 |
| eGFR10 | 0.185 | 0.146-0.225 | 0.020 | 0.000 | 0.000 | 0.000 | 1.000 |

eGFR, estimated glomerular filtration rate; SE, Std. Error; YI, youden index; Sen,sensitivity; Spe, specificity.

Table S31 ROC curve analysis of eGFR equations based on rGFR＜90 mL/min·1.73m^2^ in sexual groups

| Index | Group | eGFR1 | eGFR2 | eGFR3 | eGFR4 | eGFR5 | eGFR6 | eGFR7 | eGFR8 | eGFR9 | eGFR10 |
| --- | --- | --- | --- | --- | --- | --- | --- | --- | --- | --- | --- |
| AUC | male | 0.179 | 0.571 | 0.471 | 0.471 | 0.518 | 0.521 | 0.514 | 0.512 | 0.198 | 0.199 |
|  | female | 0.183 | 0.452 | 0.540 | 0.540 | 0.469 | 0.460 | 0.486 | 0.428 | 0.165 | 0.166 |
| 95%CI | male | 0.125-0.233 | 0.495-0.647 | 0.405-0.538 | 0.405-0.538 | 0.446-0.589 | 0.449-0.593 | 0.442-0.585 | 0.441-0.584 | 0.143-0.254 | 0.143-0.255 |
|  | female | 0.128-0.237 | 0.379-0.526 | 0.458-0.623 | 0.458-0.623 | 0.394-0.544 | 0.386-0.534 | 0.410-0.562 | 0.354-0.503 | 0.112-0.218 | 0.113-0.219 |
| SE | male | 0.028 | 0.039 | 0.034 | 0.034 | 0.037 | 0.037 | 0.037 | 0.036 | 0.028 | 0.029 |
|  | female | 0.028 | 0.038 | 0.042 | 0.042 | 0.038 | 0.038 | 0.039 | 0.038 | 0.027 | 0.027 |
| *p*-Value | male | 0.000 | 0.053 | 0.435 | 0.435 | 0.629 | 0.566 | 0.71 | 0.738 | 0.000 | 0.000 |
|  | female | 0.000 | 0.24 | 0.318 | 0.318 | 0.447 | 0.318 | 0.73 | 0.077 | 0.000 | 0.000 |
| YI | male | 0.003 | 0.156 | 0.047 | 0.047 | 0.092 | 0.081 | 0.063 | 0.062 | 0.003 | 0.003 |
|  | female | 0.000 | 0.051 | 0.13 | 0.13 | 0.037 | 0.054 | 0.053 | 0.046 | 0.000 | 0.000 |
| Sen | male | 0.003 | 0.703 | 0.274 | 0.274 | 0.732 | 0.548 | 0.583 | 0.636 | 0.003 | 0.003 |
|  | female | 0.000 | 0.178 | 0.781 | 0.781 | 0.275 | 0.134 | 0.1 | 0.078 | 0.000 | 0.000 |
| Spe | male | 1.000 | 0.453 | 0.773 | 0.773 | 0.36 | 0.533 | 0.48 | 0.427 | 1.000 | 1.000 |
|  | female | 1.000 | 0.873 | 0.349 | 0.349 | 0.762 | 0.921 | 0.952 | 0.968 | 1.000 | 1.000 |

eGFR, estimated glomerular filtration rate; SE, Std. Error; YI, youden index; Sen,sensitivity; Spe, specificity.

Table S32 ROC curve analysis of eGFR equations based on rGFR＜90 mL/min·1.73m^2^ in different age groups

| Index | Group | eGFR1 | eGFR2 | eGFR3 | eGFR4 | eGFR5 | eGFR6 | eGFR7 | eGFR8 | eGFR9 | eGFR10 |
| --- | --- | --- | --- | --- | --- | --- | --- | --- | --- | --- | --- |
| AUC | 18-39 years | 0.207 | 0.508 | 0.540 | 0.540 | 0.491 | 0.512 | 0.478 | 0.491 | 0.261 | 0.262 |
|  | 40-60 years | 0.226 | 0.506 | 0.510 | 0.510 | 0.525 | 0.52 | 0.531 | 0.514 | 0.223 | 0.224 |
|  | ＞60 years | 0.266 | 0.547 | 0.509 | 0.509 | 0.431 | 0.445 | 0.442 | 0.385 | 0.448 | 0.277 |
| 95%CI | 18-39 years | 0.099-0.315 | 0.369-0.647 | 0.400-0.680 | 0.400-0.680 | 0.352-0.630 | 0.373-0.651 | 0.338-0.617 | 0.352-0.630 | 0.144-0.379 | 0.145-0.380 |
|  | 40-60 years | 0.170-0.281 | 0.433-0.579 | 0.441-0.580 | 0.441-0.580 | 0.456-0.595 | 0.451-0.590 | 0.461-0.601 | 0.443-0.585 | 0.169-0.277 | 0.170-0.279 |
|  | ＞60 years | 0.157-0.376 | 0.417-0.677 | 0.373-0.645 | 0.373-0.645 | 0.316-0.546 | 0.331-0.558 | 0.322-0.562 | 0.273-0.497 | 0.329-0.567 | 0.160-0.395 |
| SE | 18-39 years | 0.055 | 0.071 | 0.071 | 0.071 | 0.071 | 0.071 | 0.071 | 0.071 | 0.060 | 0.060 |
|  | 40-60 years | 0.028 | 0.037 | 0.035 | 0.035 | 0.036 | 0.036 | 0.036 | 0.036 | 0.028 | 0.028 |
|  | ＞60 years | 0.056 | 0.066 | 0.069 | 0.069 | 0.059 | 0.058 | 0.061 | 0.057 | 0.061 | 0.060 |
| p-Value | 18-39 years | 0.000 | 0.907 | 0.568 | 0.568 | 0.897 | 0.868 | 0.754 | 0.897 | 0.001 | 0.001 |
|  | 40-60 years | 0.000 | 0.875 | 0.781 | 0.781 | 0.484 | 0.575 | 0.395 | 0.701 | 0.000 | 0.000 |
|  | ＞60 years | 0.000 | 0.462 | 0.882 | 0.882 | 0.281 | 0.388 | 0.364 | 0.072 | 0.412 | 0.000 |
| YI | 18-39 years | 0.000 | 0.119 | 0.199 | 0.199 | 0.097 | 0.085 | 0.127 | 0.068 | 0.000 | 0.000 |
|  | 40-60 years | 0.000 | 0.074 | 0.099 | 0.099 | 0.096 | 0.067 | 0.086 | 0.075 | 0.004 | 0.004 |
|  | ＞60 years | 0.007 | 0.150 | 0.126 | 0.126 | 0.054 | 0.075 | 0.032 | 0.061 | 0.063 | 0.000 |
| Sen | 18-39 years | 0.000 | 0.576 | 0.485 | 0.485 | 0.697 | 0.485 | 0.727 | 0.697 | 0.000 | 0.000 |
|  | 40-60 years | 0.000 | 0.901 | 0.322 | 0.322 | 0.442 | 0.166 | 0.604 | 0.346 | 0.004 | 0.004 |
|  | ＞60 years | 0.007 | 0.422 | 0.899 | 0.899 | 0.054 | 0.348 | 0.078 | 0.061 | 0.108 | 0.000 |
| Spe | 18-39 years | 1.000 | 0.543 | 0.714 | 0.714 | 0.400 | 0.600 | 0.400 | 0.371 | 1.000 | 1.000 |
|  | 40-60 years | 1.000 | 0.173 | 0.778 | 0.778 | 0.654 | 0.901 | 0.481 | 0.728 | 1.000 | 1.000 |
|  | ＞60 years | 1.000 | 0.727 | 0.227 | 0.227 | 1.000 | 0.727 | 0.955 | 1.000 | 0.955 | 1.000 |

eGFR, estimated glomerular filtration rate; SE, Std. Error; YI, youden index; Sen,sensitivity; Spe, specificity.

Table S33 ROC curve analysis of eGFR equations based on rGFR＜90 mL/min·1.73m^2^ in different weight groups

| Index | Group | eGFR1 | eGFR2 | eGFR3 | eGFR4 | eGFR5 | eGFR6 | eGFR7 | eGFR8 | eGFR9 | eGFR10 |
| --- | --- | --- | --- | --- | --- | --- | --- | --- | --- | --- | --- |
| AUC | Under weight | 0.193 | 0.614 | 0.536 | 0.536 | 0.629 | 0.639 | 0.600 | 0.400 | 0.243 | 0.243 |
|  | Normal weight | 0.154 | 0.494 | 0.498 | 0.498 | 0.529 | 0.514 | 0.543 | 0.519 | 0.171 | 0.175 |
|  | Over weight | 0.197 | 0.532 | 0.512 | 0.512 | 0.429 | 0.438 | 0.425 | 0.416 | 0.197 | 0.197 |
|  | Obesity | 0.167 | 0.535 | 0.481 | 0.481 | 0.576 | 0.575 | 0.585 | 0.514 | 0.165 | 0.166 |
| 95%CI | Under weight | -0.058-0.444 | 0.389-0.839 | 0.187-0.884 | 0.187-0.884 | 0.369-0.888 | 0.457-0.822 | 0.293-0.907 | 0.093-0.707 | -0.023-0.509 | -0.023-0.509 |
|  | Normal weight | 0.103-0.205 | 0.418-0.570 | 0.417-0.579 | 0.417-0.579 | 0.454-0.604 | 0.438-0.589 | 0.468-0.618 | 0.444-0.595 | 0.118-0.224 | 0.121-0.229 |
|  | Over weight | 0.129-0.266 | 0.439-0.624 | 0.433-0.592 | 0.433-0.592 | 0.348-0.511 | 0.355-0.521 | 0.343-0.506 | 0.336-0.497 | 0.126-0.269 | 0.126-0.268 |
|  | Obesity | 0.074-0.260 | 0.372-0.698 | 0.336-0.626 | 0.336-0.626 | 0.407-0.746 | 0.412-0.738 | 0.419-0.750 | 0.340-0.688 | 0.071-0.260 | 0.071-0.262 |
| SE | Underweight | 0.128 | 0.115 | 0.178 | 0.178 | 0.132 | 0.093 | 0.156 | 0.157 | 0.136 | 0.136 |
|  | Normal weight | 0.026 | 0.039 | 0.041 | 0.041 | 0.038 | 0.039 | 0.038 | 0.038 | 0.027 | 0.027 |
|  | Over weight | 0.035 | 0.047 | 0.041 | 0.041 | 0.042 | 0.042 | 0.042 | 0.041 | 0.036 | 0.036 |
|  | Obesity | 0.047 | 0.083 | 0.074 | 0.074 | 0.086 | 0.083 | 0.084 | 0.089 | 0.048 | 0.049 |
| p-Value | Underweight | 0.047 | 0.459 | 0.817 | 0.817 | 0.405 | 0.367 | 0.517 | 0.517 | 0.096 | 0.096 |
|  | Normal weight | 0.000 | 0.874 | 0.958 | 0.958 | 0.461 | 0.730 | 0.277 | 0.627 | 0.000 | 0.000 |
|  | Over weight | 0.000 | 0.473 | 0.784 | 0.784 | 0.111 | 0.161 | 0.089 | 0.059 | 0.000 | 0.000 |
|  | Obesity | 0.000 | 0.665 | 0.811 | 0.811 | 0.344 | 0.350 | 0.295 | 0.864 | 0.000 | 0.000 |
| YI | Underweight | 0.000 | 0.379 | 0.250 | 0.250 | 0.464 | 0.514 | 0.379 | 0.086 | 0.029 | 0.029 |
|  | Normal weight | 0.000 | 0.051 | 0.075 | 0.075 | 0.116 | 0.091 | 0.100 | 0.104 | 0.000 | 0.000 |
|  | Over weight | 0.000 | 0.113 | 0.138 | 0.138 | 0.056 | 0.025 | 0.067 | 0.072 | 0.000 | 0.000 |
|  | Obesity | 0.015 | 0.136 | 0.188 | 0.188 | 0.217 | 0.202 | 0.268 | 0.206 | 0.000 | 0.000 |
| Sen | Underweight | 0.000 | 0.629 | 1.000 | 1.000 | 0.714 | 0.514 | 0.629 | 0.086 | 0.029 | 0.029 |
|  | Normal weight | 0.000 | 0.112 | 0.802 | 0.802 | 0.313 | 0.424 | 0.252 | 0.317 | 0.000 | 0.000 |
|  | Over weight | 0.000 | 0.671 | 0.273 | 0.273 | 0.056 | 0.987 | 0.087 | 0.091 | 0.000 | 0.000 |
|  | Obesity | 0.015 | 0.574 | 0.250 | 0.250 | 0.779 | 0.765 | 0.706 | 0.706 | 0.000 | 0.000 |
| Spe | Underweight | 1.000 | 0.750 | 0.250 | 0.250 | 0.750 | 1.000 | 0.750 | 1.000 | 1.000 | 1.000 |
|  | Normal weight | 1.000 | 0.939 | 0.273 | 0.273 | 0.803 | 0.667 | 0.848 | 0.788 | 1.000 | 1.000 |
|  | Over weight | 1.000 | 0.442 | 0.865 | 0.865 | 1.000 | 0.038 | 0.981 | 0.981 | 1.000 | 1.000 |
|  | Obesity | 1.000 | 0.563 | 0.938 | 0.938 | 0.438 | 0.438 | 0.563 | 0.500 | 1.000 | 1.000 |

eGFR, estimated glomerular filtration rate; SE, Std. Error; YI, youden index; Sen,sensitivity; Spe, specificity.

Table S34 ROC curve analysis of eGFR equations based on rGFR ＜60 mL/min·1.73m^2^ in 750 participants

| Equation | AUC | 95%CI | SE | p-Value | YI | Sen | Spe |
| --- | --- | --- | --- | --- | --- | --- | --- |
| eGFR1 | 0.179 | 0.148-0.210 | 0.016 | 0.000 | 0.000 | 0.000 | 1.000 |
| eGFR2 | 0.517 | 0.474-0.559 | 0.022 | 0.441 | 0.05 | 0.208 | 0.842 |
| eGFR3 | 0.492 | 0.450-0.535 | 0.022 | 0.725 | 0.030 | 0.958 | 0.073 |
| eGFR4 | 0.492 | 0.450-0.535 | 0.022 | 0.725 | 0.030 | 0.958 | 0.073 |
| eGFR5 | 0.481 | 0.438-0.524 | 0.022 | 0.386 | 0.024 | 0.148 | 0.876 |
| eGFR6 | 0.475 | 0.433-0.518 | 0.022 | 0.256 | 0.008 | 0.032 | 0.976 |
| eGFR7 | 0.486 | 0.443-0.528 | 0.022 | 0.510 | 0.021 | 0.640 | 0.381 |
| eGFR8 | 0.476 | 0.433-0.519 | 0.022 | 0.275 | 0.031 | 0.095 | 0.936 |
| eGFR9 | 0.172 | 0.141-0.202 | 0.016 | 0.000 | 0.002 | 1.000 | 0.002 |
| eGFR10 | 0.173 | 0.143-0.204 | 0.016 | 0.000 | 0.002 | 1.000 | 0.002 |

eGFR, estimated glomerular filtration rate; SE, Std. Error; YI, youden index; Sen,sensitivity; Spe, specificity.

Table S35 ROC curve analysis of eGFR equations based on rGFR＜60 mL/min·1.73m^2^ in sexual groups

| Index | Group | eGFR1 | eGFR2 | eGFR3 | eGFR4 | eGFR5 | eGFR6 | eGFR7 | eGFR8 | eGFR9 | eGFR10 |
| --- | --- | --- | --- | --- | --- | --- | --- | --- | --- | --- | --- |
| AUC | male | 0.175 | 0.534 | 0.502 | 0.502 | 0.527 | 0.523 | 0.530 | 0.530 | 0.169 | 0.171 |
|  | female | 0.185 | 0.496 | 0.479 | 0.479 | 0.425 | 0.413 | 0.432 | 0.406 | 0.176 | 0.178 |
| 95%CI | male | 0.135-0.216 | 0.478-0.590 | 0.446-0.558 | 0.446-0.558 | 0.470-0.584 | 0.466-0.580 | 0.473-0.587 | 0.473-0.588 | 0.129-0.209 | 0.131-0.211 |
|  | female | 0.138-0.233 | 0.430-0.562 | 0.414-0.543 | 0.414-0.543 | 0.361-0.490 | 0.349-0.476 | 0.368-0.496 | 0.343-0.469 | 0.129-0.224 | 0.130-0.226 |
| SE | male | 0.021 | 0.029 | 0.029 | 0.029 | 0.029 | 0.029 | 0.029 | 0.029 | 0.02 | 0.021 |
|  | female | 0.024 | 0.034 | 0.033 | 0.033 | 0.033 | 0.032 | 0.033 | 0.032 | 0.024 | 0.024 |
| p-Value | male | 0.000 | 0.237 | 0.941 | 0.941 | 0.352 | 0.427 | 0.298 | 0.291 | 0.000 | 0.000 |
|  | female | 0.000 | 0.905 | 0.518 | 0.518 | 0.025 | 0.008 | 0.040 | 0.005 | 0.000 | 0.000 |
| YI | male | 0.000 | 0.076 | 0.042 | 0.042 | 0.087 | 0.060 | 0.087 | 0.084 | 0.004 | 0.004 |
|  | female | 0.000 | 0.077 | 0.03 | 0.03 | 0.005 | 0.000 | 0.005 | 0.005 | 0.000 | 0.000 |
| Sen | male | 0.000 | 0.711 | 0.892 | 0.892 | 0.476 | 0.524 | 0.663 | 0.223 | 1.000 | 1.000 |
|  | female | 0.000 | 0.231 | 0.974 | 0.974 | 1.000 | 0.000 | 1.000 | 1.000 | 0.000 | 0.000 |
| Spe | male | 1.000 | 0.365 | 0.151 | 0.151 | 0.611 | 0.536 | 0.425 | 0.861 | 0.004 | 0.004 |
|  | female | 1.000 | 0.847 | 0.056 | 0.056 | 0.005 | 1.000 | 0.005 | 0.005 | 1.000 | 1.000 |

eGFR, estimated glomerular filtration rate; SE, Std. Error; YI, youden index; Sen,sensitivity; Spe, specificity.

Table S36 ROC curve analysis of eGFR equations based on rGFR＜60 mL/min·1.73m^2^ in different age groups

| Index | Group | eGFR1 | eGFR2 | eGFR3 | eGFR4 | eGFR5 | eGFR6 | eGFR7 | eGFR8 | eGFR9 | eGFR10 |
| --- | --- | --- | --- | --- | --- | --- | --- | --- | --- | --- | --- |
| AUC | 18-39 years | 0.188 | 0.497 | 0.586 | 0.586 | 0.510 | 0.486 | 0.545 | 0.525 | 0.545 | 0.271 |
|  | 40-60 years | 0.192 | 0.494 | 0.491 | 0.491 | 0.520 | 0.515 | 0.519 | 0.532 | 0.171 | 0.174 |
|  | ＞60 years | 0.219 | 0.537 | 0.487 | 0.487 | 0.446 | 0.45 | 0.449 | 0.437 | 0.212 | 0.213 |
| 95%CI | 18-39 years | 0.035-0.341 | 0.329-0.664 | 0.445-0.728 | 0.445-0.728 | 0.315-0.706 | 0.297-0.676 | 0.338-0.751 | 0.325-0.725 | 0.335-0.755 | 0.115-0.427 |
|  | 40-60 years | 0.143-0.241 | 0.428-0.559 | 0.425-0.556 | 0.425-0.556 | 0.454-0.586 | 0.448-0.581 | 0.454-0.584 | 0.465-0.599 | 0.125-0.217 | 0.127-0.221 |
|  | ＞60 years | 0.169-0.270 | 0.474-0.601 | 0.423-0.550 | 0.423-0.550 | 0.383-0.509 | 0.387-0.514 | 0.385-0.512 | 0.374-0.501 | 0.162-0.261 | 0.163-0.263 |
| SE | 18-39 years | 0.078 | 0.085 | 0.072 | 0.072 | 0.100 | 0.097 | 0.105 | 0.102 | 0.107 | 0.080 |
|  | 40-60 years | 0.025 | 0.033 | 0.033 | 0.033 | 0.034 | 0.034 | 0.033 | 0.034 | 0.024 | 0.024 |
|  | ＞60 years | 0.026 | 0.032 | 0.033 | 0.033 | 0.032 | 0.032 | 0.032 | 0.032 | 0.025 | 0.025 |
|  | 18-39 years | 0.002 | 0.972 | 0.387 | 0.387 | 0.917 | 0.89 | 0.653 | 0.802 | 0.653 | 0.021 |
|  | 40-60 years | 0.000 | 0.846 | 0.781 | 0.781 | 0.557 | 0.662 | 0.566 | 0.332 | 0.000 | 0.000 |
|  | ＞60 years | 0.000 | 0.252 | 0.682 | 0.682 | 0.097 | 0.126 | 0.115 | 0.053 | 0.000 | 0.000 |
| YI | 18-39 years | 0.000 | 0.224 | 0.331 | 0.331 | 0.162 | 0.09 | 0.245 | 0.183 | 0.193 | 0.000 |
|  | 40-60 years | 0.000 | 0.034 | 0.043 | 0.043 | 0.093 | 0.079 | 0.077 | 0.103 | 0.004 | 0.004 |
|  | ＞60 years | 0.000 | 0.094 | 0.037 | 0.037 | 0.015 | 0.015 | 0.001 | 0.011 | 0.000 | 0.000 |
| Sen | 18-39 years | 0.000 | 1.000 | 0.900 | 0.900 | 0.300 | 0.900 | 0.400 | 0.200 | 0.400 | 0.000 |
|  | 40-60 years | 0.000 | 0.104 | 0.981 | 0.981 | 0.519 | 0.660 | 0.670 | 0.491 | 1.000 | 1.000 |
|  | ＞60 years | 0.000 | 0.359 | 0.898 | 0.898 | 0.922 | 0.042 | 0.928 | 0.024 | 0.000 | 0.000 |
| Spe | 18-39 years | 1.000 | 0.224 | 0.431 | 0.431 | 0.862 | 0.190 | 0.845 | 0.983 | 0.793 | 1.000 |
|  | 40-60 years | 1.000 | 0.930 | 0.062 | 0.062 | 0.574 | 0.419 | 0.407 | 0.612 | 0.004 | 0.004 |
|  | ＞60 years | 1.000 | 0.735 | 0.139 | 0.139 | 0.093 | 0.974 | 0.073 | 0.987 | 1.000 | 1.000 |

eGFR, estimated glomerular filtration rate; SE, Std. Error; YI, youden index; Sen,sensitivity; Spe, specificity.

Table S37 ROC curve analysis of eGFR equations based on rGFR ＜60 mL/min·1.73m^2^ in different weight groups

| Index | Group | | eGFR1 | eGFR2 | eGFR3 | eGFR4 | eGFR5 | eGFR6 | eGFR7 | eGFR8 | eGFR9 | eGFR10 |
| --- | --- | --- | --- | --- | --- | --- | --- | --- | --- | --- | --- | --- |
| AUC | Underweight | 0.150 | | 0.567 | 0.439 | 0.439 | 0.660 | 0.652 | 0.647 | 0.612 | 0.136 | 0.139 |
|  | Normal weight | 0.147 | | 0.470 | 0.510 | 0.510 | 0.486 | 0.493 | 0.492 | 0.496 | 0.160 | 0.162 |
|  | Overweight | 0.186 | | 0.549 | 0.479 | 0.479 | 0.428 | 0.416 | 0.432 | 0.434 | 0.191 | 0.193 |
|  | Obesity | 0.158 | | 0.57 | 0.491 | 0.491 | 0.566 | 0.535 | 0.575 | 0.497 | 0.174 | 0.173 |
| 95%CI | Underweight | 0.032-0.267 | | 0.384-0.750 | 0.251-0.626 | 0.251-0.626 | 0.486-0.835 | 0.479-0.826 | 0.471-0.823 | 0.433-0.791 | 0.022-0.251 | 0.025-0.253 |
|  | Normal weight | 0.105-0.189 | | 0.407-0.534 | 0.448-0.573 | 0.448-0.573 | 0.422-0.551 | 0.428-0.557 | 0.429-0.556 | 0.432-0.560 | 0.115-0.204 | 0.116-0.207 |
|  | Overweight | 0.136-0.237 | | 0.480-0.618 | 0.409-0.549 | 0.409-0.549 | 0.360-0.497 | 0.349-0.484 | 0.363-0.501 | 0.364-0.504 | 0.140-0.242 | 0.142-0.243 |
|  | Obesity | 0.064-0.251 | | 0.441-0.700 | 0.362-0.619 | 0.362-0.619 | 0.441-0.691 | 0.409-0.662 | 0.450-0.699 | 0.373-0.621 | 0.078-0.270 | 0.080-0.267 |
| SE | Underweight | 0.060 | | 0.093 | 0.096 | 0.096 | 0.089 | 0.089 | 0.090 | 0.091 | 0.058 | 0.058 |
|  | Normal weight | 0.021 | | 0.032 | 0.032 | 0.032 | 0.033 | 0.033 | 0.033 | 0.033 | 0.023 | 0.023 |
|  | Overweight | 0.026 | | 0.035 | 0.036 | 0.036 | 0.035 | 0.034 | 0.035 | 0.036 | 0.026 | 0.026 |
|  | Obesity | 0.048 | | 0.066 | 0.065 | 0.065 | 0.064 | 0.065 | 0.064 | 0.063 | 0.049 | 0.048 |
| p-Value | Underweight | 0.000 | | 0.479 | 0.515 | 0.515 | 0.089 | 0.106 | 0.119 | 0.234 | 0.000 | 0.000 |
|  | Normal weight | 0.000 | | 0.359 | 0.754 | 0.754 | 0.675 | 0.820 | 0.816 | 0.904 | 0.000 | 0.000 |
|  | Overweight | 0.000 | | 0.165 | 0.557 | 0.557 | 0.042 | 0.018 | 0.054 | 0.062 | 0.000 | 0.000 |
|  | Obesity | 0.000 | | 0.284 | 0.886 | 0.886 | 0.315 | 0.591 | 0.256 | 0.967 | 0.000 | 0.000 |
| YI | Underweight | 0.000 | | 0.174 | 0.099 | 0.099 | 0.374 | 0.329 | 0.329 | 0.310 | 0.000 | 0.000 |
|  | Normal weight | 0.000 | | 0.017 | 0.076 | 0.076 | 0.079 | 0.037 | 0.061 | 0.057 | 0.000 | 0.000 |
|  | Overweight | 0.000 | | 0.118 | 0.040 | 0.040 | 0.017 | 0.011 | 0.017 | 0.049 | 0.000 | 0.000 |
|  | Obesity | 0.019 | | 0.196 | 0.095 | 0.095 | 0.194 | 0.232 | 0.189 | 0.181 | 0.019 | 0.019 |
| Sen | Underweight | 0.000 | | 0.765 | 0.235 | 0.235 | 0.647 | 0.647 | 0.647 | 0.765 | 0.000 | 0.000 |
|  | Normal weight | 0.000 | | 0.197 | 0.606 | 0.606 | 0.213 | 0.055 | 0.213 | 0.150 | 0.000 | 0.000 |
|  | Overweight | 0.000 | | 0.593 | 0.880 | 0.880 | 1.000 | 1.000 | 1.000 | 0.083 | 0.000 | 0.000 |
|  | Obesity | 1.000 | | 0.290 | 0.774 | 0.774 | 0.968 | 0.968 | 0.774 | 0.935 | 1.000 | 1.000 |
| Spe | Underweight | 1.000 | | 0.409 | 0.864 | 0.864 | 0.727 | 0.682 | 0.682 | 0.545 | 1.000 | 1.000 |
|  | Normal weight | 1.000 | | 0.820 | 0.470 | 0.470 | 0.866 | 0.982 | 0.848 | 0.908 | 1.000 | 1.000 |
|  | Overweight | 1.000 | | 0.526 | 0.160 | 0.160 | 0.017 | 0.011 | 0.017 | 0.966 | 1.000 | 1.000 |
|  | Obesity | 0.019 | | 0.906 | 0.321 | 0.321 | 0.226 | 0.264 | 0.415 | 0.245 | 0.019 | 0.019 |

eGFR, estimated glomerular filtration rate; SE, Std. Error; YI, youden index; Sen,sensitivity; Spe, specificity.

| Table S38 The correlation between indicators and rGFR | | | | | | | | | | |
| --- | --- | --- | --- | --- | --- | --- | --- | --- | --- | --- |
|  |  | Sex | Age | Height | Weight | BMI | SBP | DBP | Cys C | Scr |
| rGFR | Coefficient | -0.029 | -0.367 | 0.081 | 0.075 | 0.031 | -0.159 | -0.004 | -0.635 | -0.512 |
|  | *p*-Value | 0.441 | 0.000 | 0.028 | 0.044 | 0.403 | 0.000 | 0.919 | 0.000 | 0.000 |
| BMI, body mass index (calculated as weight/height^2^); SBP, systolic blood pressure; DBP, diastolic blood pressure; Cys C, serum cystatin C; Scr, serum creatinine; rGFR, reference glomerular filtration rate. | | | | | | | | | | |

Table S39 Stepwise and LASSO regression analysis used to screen the main indicators affecting rGFR

| Item | Stepwise regression analysis | | | |  | LASSO regression analysis | |
| --- | --- | --- | --- | --- | --- | --- | --- |
|  | γ（t） | 95% CI | p-Value | VIF |  | γ（t） | p-Value |
| Constant | 77.362 (4.615) | 44.510-110.213 | 0.000 | - |  | 81.127(4.890) | 0.000 |
| Age | -0.455 (-7.983) | -0.567--0.343 | 0.000 | 1.136 |  | -0.446(-7.916) | 0.000 |
| Height | 29.662 (3.069) | 10.717-48.607 | 0.002 | 1.042 |  | 25.843(2.702) | 0.007 |
| SBP | -0.082(-2.395) | -0.149- -0.015 | 0.017 | 1.054 |  | -0.070(-2.085) | 0.037 |
| Cys C | -16.634 (-16.306) | -18.633--14.635 | 0.000 | 1.072 |  | -16.296(-16.152) | 0.000 |
| R^2^ | 0.386 | | | |  | 0.386 | |
| Adj R^2^ | 0.383 | | | |  | 0.382 | |
| F value | F (4,724) =113.903, p=0.000 | | | |  | F (4724) =113.706, p=0.000 | |

Dependent Variable: rGFR;

D-W 0.709 in stepwiseregression analysis;

SBP, systolic blood pressure; Cys C, serum cystatin C; LRT，likelihood ratio test.

Table S40 ROC curve analysis of eGFR equations based on rGFR ＜90 or 60 mL/min·1.73m^2^

|  | rGFR ＜90 mL/min·1.73m^2^ | | | | | |  | rGFR ＜60 mL/min·1.73m^2^ | | | | |
| --- | --- | --- | --- | --- | --- | --- | --- | --- | --- | --- | --- | --- |
| Index | AUC (95%CI) | *p*-Value | | YI | Sen | Spe |  | AUC (95%CI) | *p*-Value | YI | Sen | Spe |
| Age | 0.726  (0.678-0.774) | 0.000 | 0.337 | | 0.776 | 0.561 |  | 0.676  (0.636-0.716) | 0.000 | 0.278 | 0.627 | 0.651 |
| Height | 0.451  (0.394-0.508) | 0.086 | 0.032 | | 0.975 | 0.057 |  | 0.472  (0.429-0.515) | 0.200 | 0.004 | 1.000 | 0.004 |
| SBP | 0.641  （0.588-0.693） | 0.000 | 0.243 | | 0.512 | 0.732 |  | - | - | - | - | - |
| Cys C | 0.781  （0.736-0.825） | 0.000 | 0.436 | | 0.761 | 0.675 |  | 0.829  (0.797-0.861) | 0.000 | 0.546 | 0.714 | 0.832 |
| combined diagnosis | 0.810  (0.768-0.851) | 0.000 | 0.511 | | 0.820 | 0.691 |  | 0.833  (0.802-0.864) | 0.000 | 0.549 | 0.728 | 0.821 |

BMI, body mass index (calculated as weight/height^2^); SBP, systolic blood pressure; Cys C, serum cystatin C; rGFR, reference glomerular filtration rate; YI, youden index; Sen,sensitivity; Spe, specificity.
